# Band-interface cooperative engineering of bismuth-based heterojunctions for sonodynamic-chemodynamic synergistic breast cancer therapy

**DOI:** 10.1016/j.mtbio.2025.102296

**Published:** 2025-09-06

**Authors:** Xueyu Li, Jun Du, Qingxuan Meng, Lejin Zhu, Yuqing Miao, Yuhao Li, Qing Miao

**Affiliations:** a Department of Anesthesiology, Jiading District Central Hospital Affiliated Shanghai University of Medicine and Health Sciences, Shanghai University of Medicine and Health Sciences, Shanghai, 201318, China; b School of Materials and Chemistry, Institute of Bismuth Science, Shanghai Collaborative Innovation Center of Energy Therapy for Tumors, University of Shanghai for Science and Technology, Shanghai, 200093, China

**Keywords:** Bismuth, Heterojunction, Sonodynamic therapy, Chemodynamic therapy, Immunogenic cell death

## Abstract

The clinical efficacy of sono-immunotherapy is limited by the low reactive oxygen species (ROS) yield of sonosensitizers and the antioxidant defense mechanisms within the tumor microenvironment (TME). Herein, leveraging bandgap and interfacial engineering strategies, we fabricate a bismuth-based nanoheterojunction BiF_3_:Ce-BiOI-PEG (BCOP) via an ion-exchange method. BCOP integrates efficient sono-catalytic ROS generation with TME-responsive Fenton-like catalytic activity, enabling synergistic enhancement of sonodynamic therapy (SDT) and chemodynamic therapy (CDT). Under ultrasound (US) irradiation, the BCOP heterojunction significantly boosts ROS production efficiency by utilizing its built-in electric field to drive directional carrier separation. Concurrently, the acidic TME triggers a Ce^3+^-mediated Fenton-like reaction, converting endogenous H_2_O_2_ into highly toxic hydroxyl radicals (•OH). Furthermore, dual glutathione (GSH) depletion via Bi^3+^ coordination coupled with hole (h^+^)-mediated oxidation effectively impairs the antioxidant capacity of the TME, synergistically amplifying oxidative stress-induced damage in tumor cells. In vitro cell experiments demonstrate that BCOP induces mitochondrial damage, apoptosis, and immunogenic cell death (ICD) in breast cancer cells. In vivo studies further confirm its ability to activate a systemic anti-tumor immune response and markedly inhibit tumor growth. This study provides a band-interfacial cooperative regulation strategy for multimodal tumor immunotherapy.

## Graphical abstract

**Figure:**
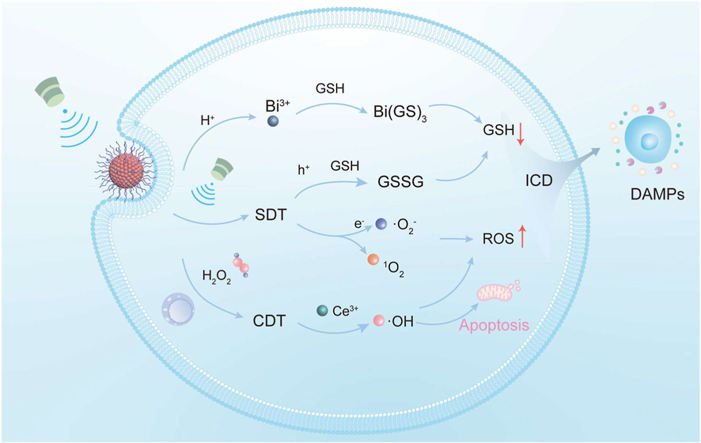

## 1 Introduction

Achieving a balance between the non-invasiveness and efficacy of tumor therapy remains a critical clinical challenge [[1], [2], [3]]. Traditional radiotherapy and chemotherapy are often plagued by systemic toxic side effects and damage to normal tissues [[4], [5], [6]]. Sonodynamic therapy (SDT), which leverages ultrasound (US)'s exceptional tissue penetration depth (>8 cm) and favorable spatiotemporal controllability [7], offers new hope for treating deep-seated tumors [8,9]. Its core mechanism involves sonosensitizers generating reactive oxygen species (ROS) upon US excitation to induce tumor cell death, rendering it a highly promising non-invasive therapeutic approach [10].

In recent years, inorganic nano-sonosensitizers have gained widespread attention due to their high chemical stability and modifiability [11,12]. Among these, semiconductor materials stand out as one of the most significant categories of inorganic nano-sonosensitizers [13]. Under US irradiation, semiconductor sonosensitizers can generate electron–hole pairs (e^−^–h^+^), which subsequently facilitate ROS production [14]. However, existing semiconductor sonosensitizers still face several critical limitations: a high carrier recombination rate that drastically reduces the number of effective charge carriers [15]; insufficient ROS yield, which severely compromises therapeutic efficacy [16]; and poor adaptability to the tumor microenvironment (TME) [17]. The TME's abundant reductive substances such as glutathione (GSH) and other antioxidant defense mechanisms efficiently scavenge ROS, further undermining SDT effectiveness [18,19]. Thus, enhancing sonocatalytic efficiency and endowing materials with TME-responsive catalytic functions are key to overcoming SDT's efficacy bottleneck.

To address these challenges, researchers have proposed various strategies. For improving sonocatalytic efficiency, modulating the semiconductor band structure is central, such as constructing heterojunctions or employing elemental doping [[20], [21], [22]]. To overcome TME-mediated antioxidant resistance, strategies primarily involve introducing specific metal ions to catalyze Fenton-like reactions for generating highly oxidizing hydroxyl radicals (•OH) or designing materials to actively deplete GSH [[23], [24], [25]]. However, these approaches often only partially mitigate the issues; they struggle to synergistically enhance sonocatalytic efficiency and TME responsiveness, nor do they effectively activate immunogenic cell death (ICD). For instance, while heterojunction construction promotes carrier separation, it exerts limited impact on the TME's complex antioxidant systems, making it difficult to accumulate sufficient ROS to trigger ICD [26,27]. Conversely, GSH-depletion-focused strategies cannot fully meet the demand for high-efficiency sonocatalytic ROS generation, nor can they provide the robust oxidative stress required to initiate ICD [28,29].

Notably, efficient ROS generation is not only fundamental to SDT's direct tumor-killing effect but also critical for activating anti-tumor immune responses and triggering ICD [[30], [31], [32]]. ROS-mediated endoplasmic reticulum stress can induce the release of damage-associated molecular patterns (DAMPs) such as calreticulin (CRT), high-mobility group box 1 (HMGB1), and adenosine triphosphate (ATP) from tumor cells. This drives the maturation of antigen-presenting cells, activates antigen-specific cytotoxic T lymphocyte responses, and establishes a robust adaptive anti-tumor immune response [[33], [34], [35], [36]]. Consequently, an ideal sonosensitizer must integrate multiple synergistic functions: highly efficient sonocatalytic ROS generation, intelligent TME responsiveness/modulation, and ICD activation. Only through such synergy can the current limitations of SDT be truly overcome, enabling substantial improvements in tumor therapeutic outcomes.

**Scheme 1:**
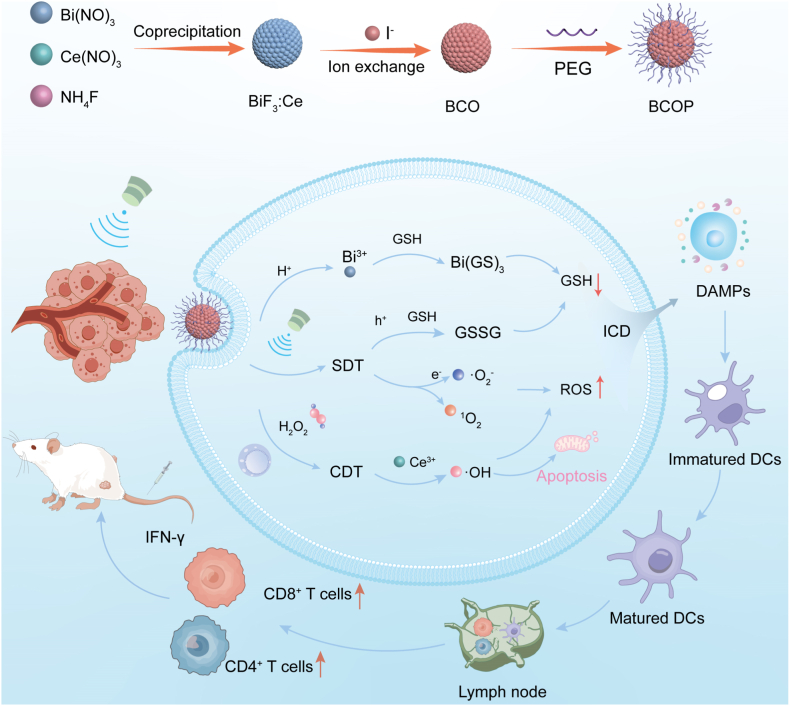
The synthesis process of BCOP and its sonodynamic-chemodynamic synergistic immunostimulatory therapeutic mechanism.

Bismuth (Bi)-based biomaterials have attracted considerable interest in biomedicine due to their excellent biocompatibility and low toxicity, with various Bi-based nanostructures demonstrating efficient US-triggered ROS generation capabilities [37]. Building on previous findings regarding the specific coordination between Bi^3+^ and GSH [38] and combining this with rare-earth element doping to modulate band structures, we proposed an approach: constructing a bismuth-based heterojunction, BiF_3_:Ce-BiOI-PEG (BCOP), via ion-exchange-based band-interface cooperative engineering (Scheme 1). This design achieves Ce^3+^ doping to modulate the band structure of BiF_3_ and utilizes the partial substitution of F^−^ in BiF_3_:Ce by I^−^ to enable in-situ growth of a BiOI layer on the nanoparticle surface, forming a BiF_3_:Ce-BiOI (BCO) heterojunction with an intimate interface. The heterojunction significantly promotes carrier separation and enhances ROS generation efficiency. We developed a sonodynamic-chemodynamic synergistic therapy by integrating dual GSH depletion (Bi^3+^ coordination coupled with h^+^-mediated oxidation) with Ce^3+^-mediated Fenton-like reactions, maximizing oxidative stress in tumor cells. The resultant ROS burst not only induces cell apoptosis but also effectively triggers ICD, thereby activating a systemic anti-tumor immune response. This study not only provides an efficient and safe synergistic immunotherapy nanoplatform but also offers a strategy for designing multifunctional heterojunction sonosensitizers.

## 2 Results and discussion

### 2.1 Synthesis and characterization of BCOP

The BiF_3_:Ce-BiOI (BCO) heterojunction was constructed using a two-step strategy involving coprecipitation followed by ion exchange. First, the bismuth-based semiconductor BiF_3_ was prepared via a coprecipitation method. Rare-earth Ce^3+^ was introduced to modulate its semiconductor properties, yielding BiF_3_:Ce. Characterization results showed that as the Ce^3+^ doping ratio increased (5%, 10%, 15%, 20%), the particle size of BiF_3_:Ce gradually decreased from 80 nm to 20 nm, and the morphology evolved into regular spheres (Fig. S1A–D). To investigate the effect of Ce^3+^ doping on the sonocatalytic performance of BiF_3_, 1,3-diphenylisobenzofuran (DPBF) was employed as a ROS probe to monitor its degradation efficiency under US irradiation. The results demonstrated that as the Ce^3+^ doping ratio increased, the attenuation rate of the characteristic DPBF absorption peak at 424 nm positively correlated with the doping concentration, confirming that ROS generation efficiency was significantly enhanced with higher doping levels (Fig. S2A–E). This finding verifies that Ce^3+^ doping effectively improves the ROS-producing capability of BiF_3_ in sonocatalytic processes. Given that BiF_3_:20%Ce exhibited the optimal ROS generation capability among Ce-doped BiF_3_, a 20% Ce doping ratio was selected for subsequent studies.

**Fig. 1:**
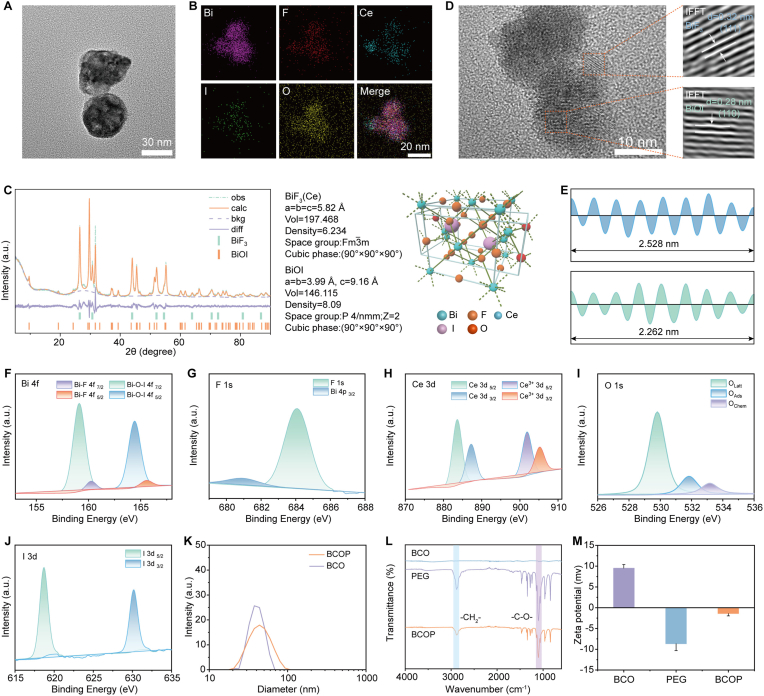
Structural characterization of BCO and BCOP. (A) TEM image of BCO, (B) EDS mapping of BCO, (C) XRD pattern and Rietveld refinement results of BCO, (D) HRTEM image of BCO (inset shows lattice fringes processed by IFFT), (E) lattice spacings derived from HRTEM images of BCO. High-resolution XPS spectra of elements in BCO: (F) Bi 4f, (G) F 1s, (H) Ce 3d, (I) O 1s, (J) I 3d. (K) DLS profiles, (L) FTIR spectra of BCO and BCOP, and (M) Zeta potential results (Mean ± SD, n = 3).

To further enhance the sonocatalytic activity of BiF_3_:20%Ce, I^−^ was introduced into BiF_3_:20%Ce via an ion-exchange method to construct the BCO heterojunction. Transmission electron microscopy (TEM) revealed that BCO particles are spherical with an average diameter of approximately 25 nm (Fig. 1A and S3), with morphology and size similar to those of BiF_3_:Ce. Energy-dispersive X-ray spectroscopy (EDS) confirmed the homogeneous distribution of Bi, F, Ce, I, and O elements (Fig. 1B), providing visual evidence for the formation of the BCO heterojunction. In the X-ray diffraction (XRD) pattern, the diffraction peaks of BCO closely matched the characteristic peaks of standard BiF_3_ (JCPDS#51–0944) and BiOI (JCPDS#10–0445) (Fig. 1C). XRD-Rietveld whole-pattern fitting determined that the sample consisted of tetragonal BiOI (ICDS#391354, space group *P*4/nmm) and cubic BiF_3_:Ce (ICDS#24522, space group Fm $\bar{3}$ m). Semi-quantitative analysis showed that the BiOI phase accounted for 70% and the BiF_3_:Ce phase for 30%. High-resolution TEM (HRTEM) observed two distinct lattice fringes: 0.32 nm corresponding to the (111) plane of BiF_3_ and 0.28 nm corresponding to the (110) plane of BiOI (Fig. 1D and E), directly confirming the presence of the heterojunction interface.

X-ray photoelectron spectroscopy (XPS) was used to systematically analyze the chemical states of elements in BCO. The characteristic peaks in the Bi 4f spectrum indicated the coexistence of Bi in Bi–F bonds and Bi–O–I structures, reflecting the synergistic bonding within the heterojunction (Fig. 1F). The characteristic peak at 685 eV in the F 1s spectrum was assigned to the Ce–F bond, confirming successful Ce^3+^ doping and Ce–F coordination, which supports the regulatory effect of Ce on the electronic structure (Fig. 1G). The multiple splitting peaks in the Ce 3d spectrum indicated the coexistence of Ce^3+^ and Ce^4+^, reflecting the favorable redox activity of Ce that provides the driving force for charge transfer (Fig. 1H). The main peak at 530 eV in the O 1s spectrum corresponded to lattice oxygen (O_latt_), which participates in the formation of the Bi–O–I bonding network (Fig. 1I). The characteristic peaks in the I 3d spectrum demonstrated that I existed as I^−^ within the structure via Bi–O–I bonds, confirming the chemical stability of the heterojunction (Fig. 1J).

To improve the biocompatibility of BCO, amphiphilic poly(ethylene glycol) (PEG) was used for surface modification (Fig. S4A), resulting in BCOP. Dynamic light scattering (DLS) showed that the hydrodynamic diameter of BCOP was approximately 55 nm (Fig. 1K), slightly larger than the size determined by TEM. This discrepancy may be attributed to the formation of a hydration shell in water. Furthermore, DLS monitoring over seven days indicated that BCOP has good stability (Fig. S4B). The appearance of characteristic peaks for –CH_2_– and –C–O– in the Fourier transform infrared (FTIR) spectrum confirmed successful PEG modification (Fig. 1L). The change in zeta potential further verified successful PEG coating, which enhances the material's biocompatibility (Fig. 1M).

### 2.2 Investigation of sonocatalytic performance and mechanism

**Fig. 2:**
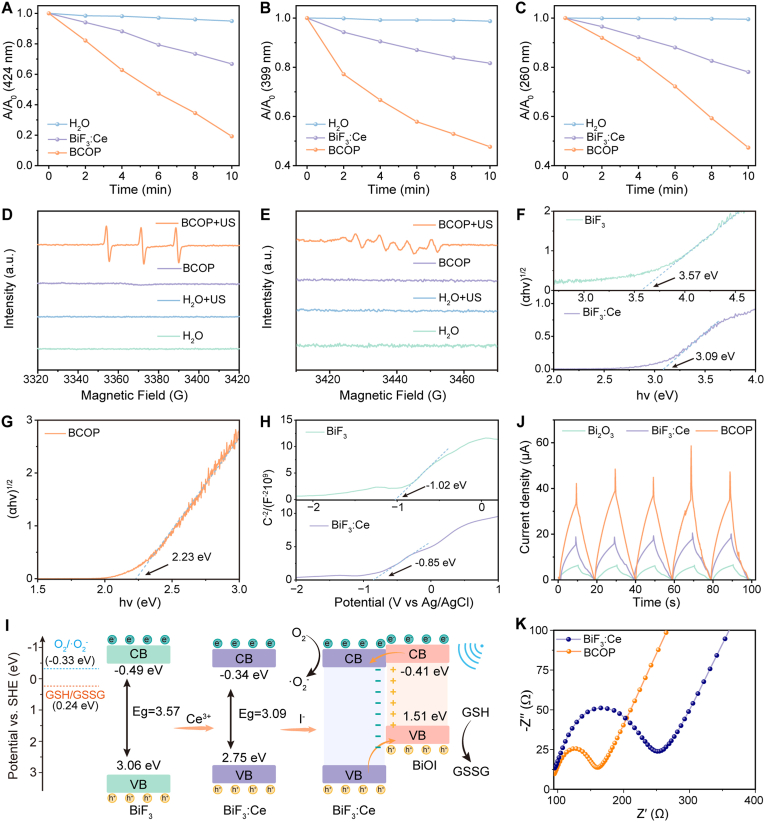
Investigation of sonocatalytic performance and mechanism of BCOP. (A) Absorbance changes of DPBF at 424 nm. (B) Absorbance changes of DPA at 399 nm. (C) Absorbance changes of NBT at 260 nm. ESR spectra of (D) ^1^O_2_ and (E) •O_2_^−^. (F) DRS results of BiF_3_ and BiF_3_:Ce. (G) DRS spectrum of BCOP. (H) Mott–Schottky plots of BiF_3_ and BiF_3_:Ce. (I) Schematic illustration of the sonocatalytic mechanism of BCOP. (J) Transient sonocurrent responses of Bi_2_O_3_, BiF_3_:Ce, and BCOP. (K) Nyquist plots of BiF_3_:Ce and BCOP.

To evaluate the sonocatalytic performance of BCOP, DPBF was employed as a ROS probe. Experimental results showed that under ultrasonic irradiation, the intensity of the characteristic absorption peak at 424 nm decreased continuously with prolonged irradiation time. After 10 min of irradiation, the amount of DPBF consumed by BCOP was approximately 2.2 times that of commercial Bi_2_O_3_ and 2.4 times that of BiF_3_:Ce (Fig. 2A, S5A–C), preliminarily indicating that constructing the BCOP heterojunction effectively enhances ROS generation efficiency. Concurrently, Fig. S5D and E revealed that GSH has a negligible influence on ROS production catalyzed by BCOP.

To further identify the specific types of ROS generated by BCOP under US catalysis, 9,10-diphenylanthracene (DPA) and nitro blue tetrazolium chloride (NBT) were used as specific probes for singlet oxygen (^1^O_2_) and superoxide anion radicals (•O_2_^−^), respectively. As shown in Fig. 2B and S6A–C, the absorption peak at 399 nm of the BCOP/DPA mixed system decreased significantly with prolonged US exposure. After 10 min, the amount of DPA consumed by BCOP was approximately 2.7 times that consumed by BiF_3_:Ce. Simultaneously, Fig. 2C and S6D–F demonstrated that the intensity of NBT's characteristic absorption peak at 260 nm also weakened under US irradiation, with the amount of NBT consumed by BCOP after 10 min being approximately 2.4 times that consumed by BiF_3_:Ce. These results collectively confirm that BCOP efficiently generates ^1^O_2_ and •O_2_^−^ under US catalysis. This conclusion was further validated by electron spin resonance (ESR) spectroscopy analysis: the typical 1:1:1 triplet signal in Fig. 2D indicated the generation of ^1^O_2_, while the characteristic signal peaks attributable to •O_2_^−^ were observed in Fig. 2E.

To deeply investigate BCOP's sonocatalytic mechanism, a systematic analysis of its band structure was performed. Diffuse reflectance spectroscopy (DRS) measurements revealed that the band gaps (E_g_) of BiF_3_, BiF_3_:Ce, and BCOP were 3.57 eV, 3.09 eV, and 2.23 eV, respectively (Fig. 2F and G). These results demonstrate that rare-earth Ce doping and subsequent heterojunction construction significantly reduce the semiconductor band gap, facilitating carrier transport. Mott–Schottky tests determined the flat-band potentials (E_fb_) of BiF_3_ and BiF_3_:Ce (vs. Ag/AgCl) to be −1.02 eV and −0.85 eV, respectively (Fig. 2H). Converting these to potentials versus the normal hydrogen electrode (SHE), using the Nernst equation [39], the calculated E_fb_ (vs. SHE) for BiF_3_ and BiF_3_:Ce were −0.39 eV and −0.24 eV, respectively. Given that both are n-type semiconductors, the conduction band (CB) positions of BiF_3_ and BiF_3_:Ce were estimated to be −0.49 eV and −0.34 eV (vs. SHE). Combined with the E_g_ values, their valence band (VB) positions were derived as 3.06 eV and 2.75 eV (vs. SHE), respectively. For comparison, the CB and VB positions of the p-type semiconductor BiOI have been reported in the literature as −0.41 eV and 1.51 eV (vs. SHE) [40].

Based on the above band structure analysis, we propose the mechanism for enhanced sonocatalysis in the BCOP heterojunction constructed via band-interface cooperative engineering (Fig. 2I). Within the BCOP heterojunction, the band alignment between BiOI and BiF_3_:Ce formed a typical type-II heterojunction structure: the CB of BiOI (−0.41 eV) is higher than that of BiF_3_:Ce (−0.34 eV), while the VB of BiOI (1.51 eV) is significantly lower than that of BiF_3_:Ce (2.75 eV). This band alignment drives the spontaneous flow of electrons from the CB of BiOI to the CB of BiF_3_:Ce until a new Fermi level equilibrium is reached. This process generates a built-in electric field at the interface directed from BiOI towards BiF_3_:Ce, causing the energy bands of BiOI to bend downwards and those of BiF_3_:Ce to bend upwards. Simultaneously, it promotes the transfer of holes from the VB of BiF_3_:Ce to the VB of BiOI, achieving effective spatial separation of photo-generated electron-hole pairs. The intimate heterojunction interface constructed via band-interface cooperative engineering further reduces the interfacial energy barrier for charge transfer, significantly enhancing interfacial charge migration efficiency. During sonocatalysis, this optimized carrier separation mechanism effectively suppresses electron–hole recombination, thereby substantially improving ROS generation efficiency and ultimately strengthening sonocatalytic performance.

Furthermore, electrochemical test results provided additional corroboration. Under US excitation, the transient sonocurrent intensity generated by BCOP increased significantly, approximately 7 times that of Bi_2_O_3_ and 2 times that of BiF_3_:Ce (Fig. 2J), directly indicating that BCOP possesses a significantly enhanced ability to generate free electrons under the acoustic field, leading to improved efficiency of ROS generation via electron capture by surrounding oxygen species. Concurrently, electrochemical impedance spectroscopy (EIS) tests showed that the Nyquist semicircle radius of BCOP was markedly smaller than that of BiF_3_:Ce (Fig. 2K), indicating lower charge transfer resistance and significantly improved carrier transport efficiency. This finding is highly consistent with the aforementioned sonocatalytic performance and band mechanism analysis.

### 2.3 Investigation of CDT performance and GSH depletion

A hallmark of the TME is its imbalanced redox homeostasis, characterized by overexpression of GSH and hydrogen peroxide (H_2_O_2_). Under the acidic conditions of the TME, Ce^3+^ in BCOP can catalyze the decomposition of H_2_O_2_ via a Fenton-like reaction, forming a Ce^3+^/Ce^4+^ redox cycle. This process not only continuously consumes H_2_O_2_ but also generates •OH, exacerbating oxidative stress within the TME and thereby achieving the cytotoxic effect of chemodynamic therapy (CDT) on tumor cells.

**Fig. 3:**
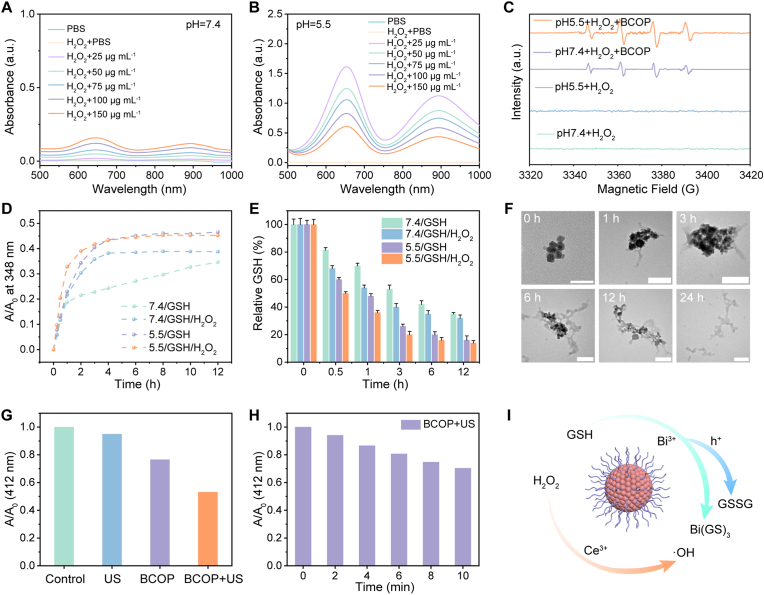
Investigation of CDT performance and GSH depletion. Absorption spectra of ox-TMB relative to BCOP concentration in (A) pH 7.4 and (B) pH 5.5 solutions. (C) ESR spectrum of •OH. (D) Time-dependent absorbance changes of BCOP supernatants at 348 nm under different conditions. (E) Relative GSH content detected by DTNB. (F) TEM images of BCOP at different time points under pH 5.5/GSH/H_2_O_2_ conditions (Scale bar: 200 nm). (G) Normalized absorbance of DTNB at 412 nm in GSH mixtures across experimental groups. (H) Time-dependent absorbance changes of DTNB at 412 nm in BCOP/GSH mixtures under US irradiation. (I) BCOP's dual GSH depletion and CDT mechanisms.

To validate this mechanism, 3,3′,5,5′-tetramethylbenzidine (TMB) was used as a •OH probe to assess BCOP's •OH generation capability under simulated physiological conditions (pH 7.4 + H_2_O_2_) and TME-mimicking conditions (pH 5.5 + H_2_O_2_). As shown in Fig. 3A and B, under TME conditions, colorless TMB was oxidized to blue ox-TMB, and its characteristic absorption peak at 652 nm increased significantly, with the enhancement exhibiting concentration dependence on BCOP. The appearance of the characteristic •OH signal (1:2:2:1) in the ESR spectrum further confirmed •OH generation (Fig. 3C), with signal intensity under TME conditions being markedly higher than under physiological conditions. These results collectively demonstrate that BCOP efficiently undergoes the Fenton-like reaction in the acidic TME, producing •OH and exerting CDT effects.

Furthermore, we investigated the GSH depletion behavior and self-degradation of BCOP. Our previous studies have indicated that Bi^3+^ in bismuth-based nanomaterials can deplete GSH and trigger material degradation by forming Bi(GS)_3_ complexes through coordination with the thiol groups of GSH. Simulated degradation experiments (Fig. 3D and S7A–D), monitored via the characteristic absorption peak of Bi(GS)_3_ at 348 nm, revealed that BCOP degradation was most pronounced under TME conditions and least significant in the pH 7.4 + GSH environment. Quantitative detection of GSH consumption using 5,5′-dithiobis (2-nitrobenzoic acid) (DTNB) further indicated that BCOP consumed the most GSH over time under TME conditions (Fig. 3E), highlighting the acidic environment and H_2_O_2_ as key factors driving sustained BCOP degradation and GSH depletion. TEM images visually demonstrated the gradual disintegration and eventual disappearance of BCOP nanoparticles over time in the TME (Fig. 3F and S8). This body of evidence fully confirms that BCOP can undergo self-degradation within the TME after exerting its CDT function.

Given that the VB position of BCOP (2.75 eV vs. SHE) satisfies the thermodynamic requirement for oxidizing GSH (0.24 eV vs. SHE) (Fig. 2I) [41], we further studied its ability to deplete GSH via the hole oxidation pathway under US excitation. As shown in Fig. 3G and S9A, after 20 min of treatment across different groups, GSH depletion in the BCOP + US group reached 47%. Moreover, GSH depletion showed a positive correlation with US irradiation time (Fig. 3H and S9B). This result indicates that in addition to depleting GSH via Bi^3+^-GSH coordination, BCOP can utilize the holes generated under US excitation to directly oxidize GSH, thereby initiating a dual GSH depletion mode (Fig. 3I).

Thus, BCOP achieves dual therapeutic effects. It effectively utilizes endogenous H_2_O_2_ in the TME to generate •OH through a Fenton-like reaction for CDT, while concurrently depleting GSH via Bi^3+^ coordination and hole oxidation pathways. This synergistic action significantly elevates intracellular oxidative stress and enhances antitumor efficacy. Furthermore, its TME-responsive self-degradation substantially improves the material's biosafety profile.

### 2.4 Evaluation of In vitro therapeutic efficacy

**Fig. 4:**
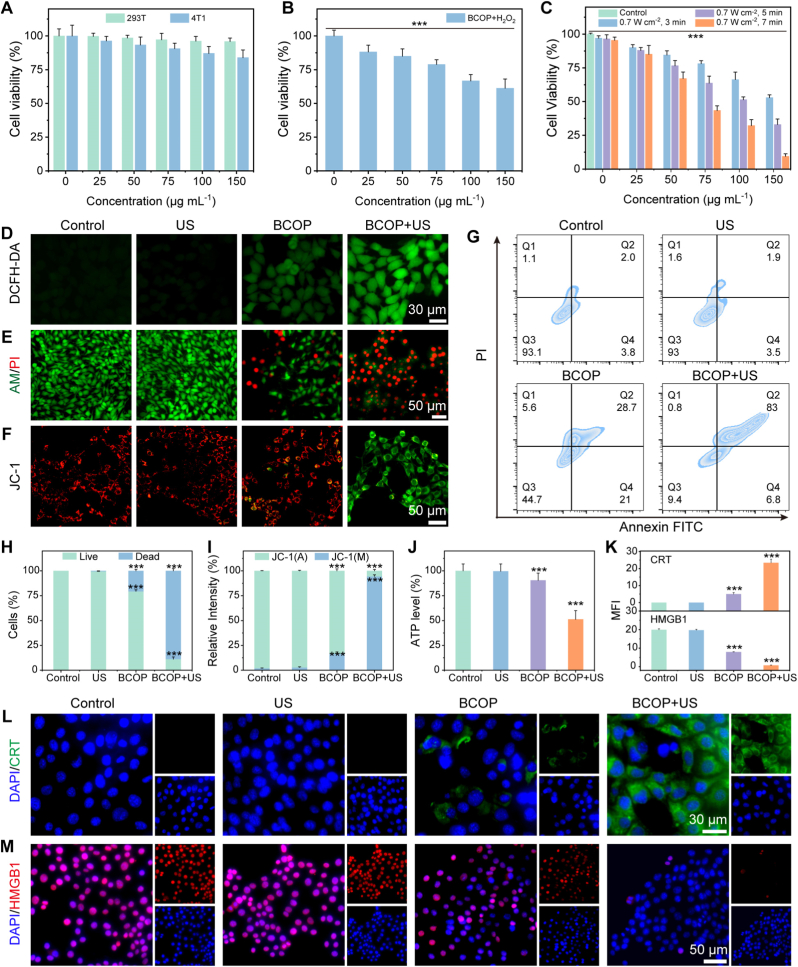
In vitro therapeutic efficacy evaluation. (A) Relative viability of 293T cells and 4T1 cells after 48 h incubation with different concentrations of BCOP (n = 5). (B) Relative viability of 4T1 cells incubated with different concentrations of BCOP in the presence of H_2_O_2_ for 48 h (n = 5). (C) Viability of 4T1 cells treated with different BCOP concentrations under varying US irradiation durations (n = 5). (D) DCFH-DA staining. (E) Calcein-AM/PI staining and (H) quantitative fluorescence analysis. (F) JC-1 staining images and (I) quantitative fluorescence analysis. (G) Apoptosis analysis by Annexin V-FITC/PI staining via flow cytometry. (J) Intracellular ATP content. (L) Immunofluorescence images of CRT and (M) HMGB1 with (K) corresponding quantitative analysis. Data are expressed as mean ± SD. ∗p < 0.05, ∗∗p < 0.01, ∗∗∗p < 0.001 (unpaired two-tailed Student's t-test).

Given BCOP's excellent performance in SDT and CDT, we further evaluated its in vitro therapeutic efficacy against 4T1 tumor cells at the cellular level. First, the biosafety of BCOP was assessed using the Cell Counting Kit-8 (CCK-8) assay. Results showed that after co-incubation with normal 293T cells for 48 h, cell viability remained above 95% even at a high BCOP concentration of 150 μg mL^−1^ (Fig. 4A), indicating no significant toxicity toward normal cells. In contrast, under the same concentration, the viability of 4T1 tumor cells decreased to 83%. This selective toxicity likely arises from the Fenton-like reaction triggered by the interaction between overexpressed H_2_O_2_ in the TME and Ce^3+^ in BCOP. To validate this mechanism, we added exogenous H_2_O_2_ to the co-incubation system and found that it significantly synergistically enhanced the cytotoxic effect of BCOP on 4T1 cells (Fig. 4B), confirming that BCOP can specifically kill tumor cells by catalyzing H_2_O_2_ to produce •OH.

Further evaluation of the sonocatalytic therapeutic effect revealed that 4T1 cell viability decreased significantly with increasing BCOP concentration and US power (Fig. 4C). When treated with 150 μg mL^−1^ BCOP combined with US irradiation (7 min), cell viability was only 9%, highlighting its remarkably efficient sonocatalytic anti-tumor activity.

To elucidate the mechanism of BCOP-mediated cell death, we employed the 2′,7′-dichlorodihydrofluorescein diacetate (DCFH-DA) probe to detect intracellular ROS levels. Fig. 4D showed that the BCOP + US group exhibited the strongest green fluorescence, indicating massive ROS generation; the BCOP group also showed weaker fluorescence, which is related to CDT. Calcein acetoxymethyl ester/propidium iodide (Calcein-AM/PI) double staining results demonstrated that cell death (red fluorescence) was most pronounced in the BCOP + US group, followed by the BCOP group (Fig. 4E and H), indicating that the ROS generated synergistically by sonodynamic and chemodynamic catalysis exerts a potent cytotoxic effect on tumor cells, with sonocatalysis contributing more prominently.

Studies have shown that ROS bursts can induce mitochondrial damage and initiate apoptotic pathways. A decrease in mitochondrial membrane potential (ΔΨm) is a hallmark of mitochondrial impairment. We assessed ΔΨm changes using the JC-1 mitochondrial membrane potential detection kit. JC-1 staining showed bright green fluorescence in the BCOP + US group (Fig. 4F and I), indicating severe mitochondrial damage; partial damage was also observed in the BCOP group. Flow cytometric apoptosis analysis revealed that the apoptosis rate in the BCOP + US group reached 83%, significantly higher than the 29% in the BCOP group (Fig. 4G), further quantitatively confirming that the synergistic therapy can efficiently induce apoptosis.

Recent studies indicate that ROS-mediated oxidative stress, besides inducing apoptosis, can also trigger ICD [42]. ICD is characterized by the release of DAMPs from dying tumor cells, including CRT translocation to the cell membrane, extracellular secretion of HMGB1, and ATP release, thereby activating anti-tumor immune responses. Based on the substantial ROS produced by BCOP under sonodynamic-chemodynamic conditions, we evaluated its ability to induce ICD. ATP detection showed intracellular ATP content decreased to 51% in the BCOP + US group (Fig. 4J). Extracellular ATP, as a key danger signal, can recruit immune cells by activating purinergic receptors on dendritic cells. Immunofluorescence results demonstrated significant CRT translocation to the cell membrane and evident HMGB1 secretion from the nucleus to the extracellular space in the BCOP + US group (Fig. 4K–M). These results collectively confirmed that BCOP-mediated sonodynamic-chemodynamic synergy can effectively induce ICD, laying the foundation for subsequent activation of anti-tumor immune responses.

### 2.5 Evaluation of In vivo therapeutic efficacy

Based on the excellent in vitro anti-tumor effects of BCOP, we further evaluated its in vivo therapeutic efficacy using a 4T1 tumor-bearing mouse model. First, the in vivo biosafety of BCOP was verified. Red blood cell hemolysis assays showed that even at a high BCOP concentration of 150 μg mL^−1^, the hemolysis rate remained below 5.0% (Fig. S10). After intravenous injection of BCOP (100 μg mL^−1^, 100 μL) for 14 days, the hematological parameters of mice were all within normal ranges (Fig. S11), and Hematoxylin and Eosin (H&E) staining of major organs showed no signs of tissue damage or inflammatory infiltration (Fig. S12), indicating good in vivo biocompatibility of BCOP. To track biodistribution, we intravenously injected near-infrared dye IR780-labeled BCOP (BCOP-IR780) into tumor-bearing mice. Fluorescence imaging revealed that the signal at the tumor site peaked at 12 h post-injection (Fig. S13A), indicating this as the optimal time window for US treatment. The blood half-life of BCOP followed the classical two-compartment model (*τ*_1/2*α*_ = 0.39 h, *τ*_1/2*β*_ = 7.97 h), a characteristic that facilitated its accumulation at tumor sites (Fig. S14). It has been reported that Bi-based nanoparticles are excreted via renal and hepatic metabolism after degradation [43,44]. Fig. S13B and C showed that the signals in major organs gradually faded over time and eventually disappeared, confirming that BCOP could be effectively metabolized.

**Fig. 5:**
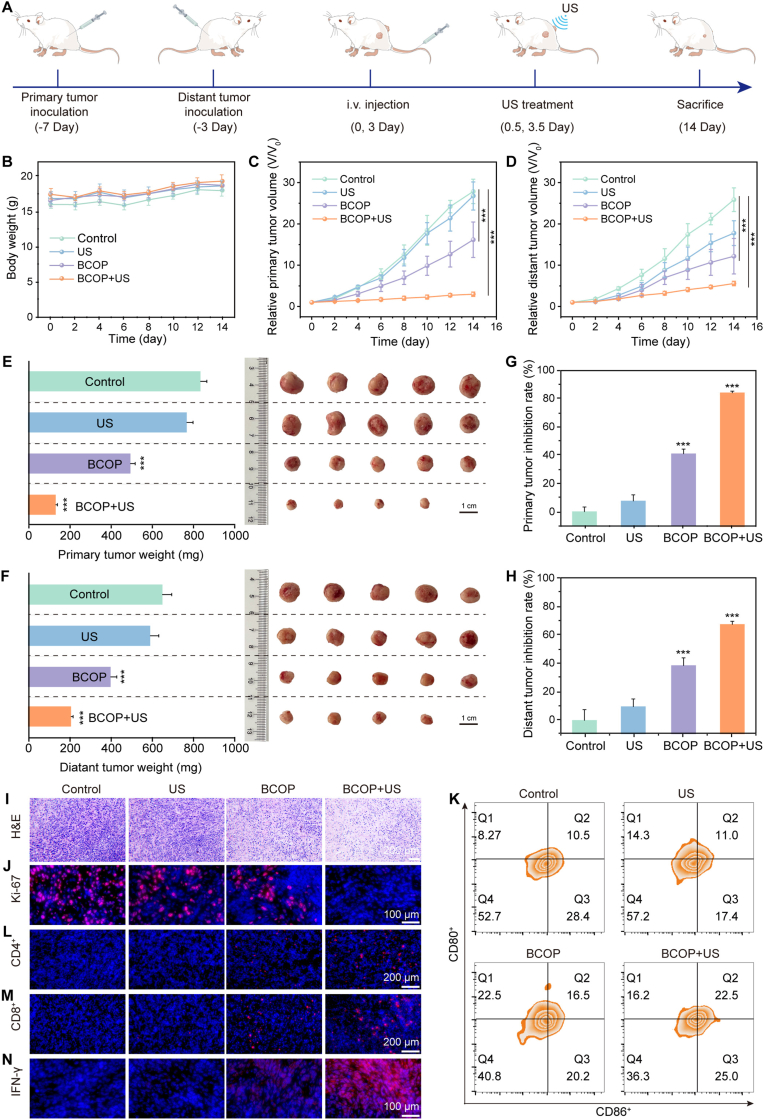
In vivo therapeutic efficacy evaluation. (A) Schematic of the treatment protocol for 4T1 tumor-bearing mice. (B) Body weight changes of mice in different treatment groups over time. Relative tumor volumes of (C) primary tumors and (D) distant tumors across groups. (E) Weights and photographs of primary tumors; (F) weights and photographs of distant tumors after 14 days of treatment. Tumor inhibition rates for (G) primary tumors and (H) distant tumors. (I) H&E staining of primary tumors. (J) Ki-67 staining of primary tumors. (K) Flow cytometric analysis of DC maturation in spleens. Immunofluorescence staining images of (L) CD4^+^ T cells, (M) CD8^+^ T cells, and (N) IFN-γ. Data are expressed as mean ± SD, n = 5. ∗p < 0.05, ∗∗p < 0.01, ∗∗∗p < 0.001 (unpaired two-tailed Student's t-test).

To assess in vivo anti-tumor efficacy, mice were randomly divided into four groups (n = 5): Control, US, BCOP, and BCOP + US. The mice then underwent the corresponding treatments (Fig. 5A). A bilateral tumor model was established to comprehensively evaluate the anti-tumor effect of BCOP. During treatment, the body weights of mice in all groups remained stable (Fig. 5B), indicating good safety of the treatment regimen. Tumor growth curves showed rapid proliferation in the Control and US groups, while both the BCOP and BCOP + US groups exerted significant inhibition on both primary and distant tumors (Fig. 5C and D). The weights and photographs of primary and distant tumors at the treatment endpoint further confirmed this conclusion (Fig. 5E and F). Compared with the primary tumor Control group, the tumor inhibition rates for the BCOP and BCOP + US groups were 41% and 84%, respectively (Fig. 5G); the inhibition rates for distant tumors were 39% and 68%, respectively (Fig. 5H), highlighting the potent tumor-inhibitory effect of the synergistic therapy.

Histological analysis further confirmed the impact of synergistic therapy on tumor cell apoptosis and proliferation. H&E staining of tumor sections showed large necrotic areas and typical apoptotic features (e.g., nuclear pyknosis) in the BCOP + US group (Fig. 5I). Ki-67 proliferation marker staining indicated partial downregulation of proliferative activity in the BCOP group, while the BCOP + US group exhibited significant suppression (Fig. 5J), confirming that the therapy functions via dual pathways of inducing apoptosis and inhibiting proliferation.

We validated the immune-activating effects induced by BCOP-mediated synergistic therapy. Recent studies indicate that assessing the maturation state of dendritic cells (DCs) and the activation level of T lymphocytes are key indicators for evaluating immune system activation [[45], [46], [47]]. Therefore, we analyzed DC maturation in tumor tissues, spleen, and lymph nodes using flow cytometry. As shown in Fig. 5K and Fig. S15, the proportion of mature DCs (CD80^+^/CD86^+^) in the BCOP + US group was significantly higher than in all control groups, demonstrating that DAMPs released by ICD effectively activated DCs. Activated mature DCs migrate to the draining lymph nodes, where they activate different subsets of T lymphocytes via MHC-I/II-antigen peptide complexes, forming the core driving network of the anti-tumor immune response. CD8^+^ T cells, upon recognizing tumor antigen-MHC I complexes via their T cell receptor (TCR), differentiate into cytotoxic T lymphocytes (CTLs). Concurrently, activated CD4^+^ T cells, following recognition of MHC II-antigen peptide complexes, secrete cytokines such as IL-2 and IFN-γ. These cytokines not only promote the clonal expansion and effector function maintenance of CD8^+^ T cells but also synergistically regulate the antigen presentation efficiency of DCs, thus playing a critical helper regulatory role in anti-tumor immunity.

Immunofluorescence results of tumor tissues showed significantly upregulated infiltration of CD4^+^ T cells and CD8^+^ T cells (Fig. 5L and M), as well as enhanced IFN-γ expression in the BCOP + US group (Fig. 5N), indicating that BCOP-based sonodynamic and chemodynamic therapy elicited an adaptive immune response, enhancing anti-tumor capability and effectively inhibiting tumor growth.

### 2.6 Biological mechanism of action of BCOP in anti-tumor therapy

**Fig. 6:**
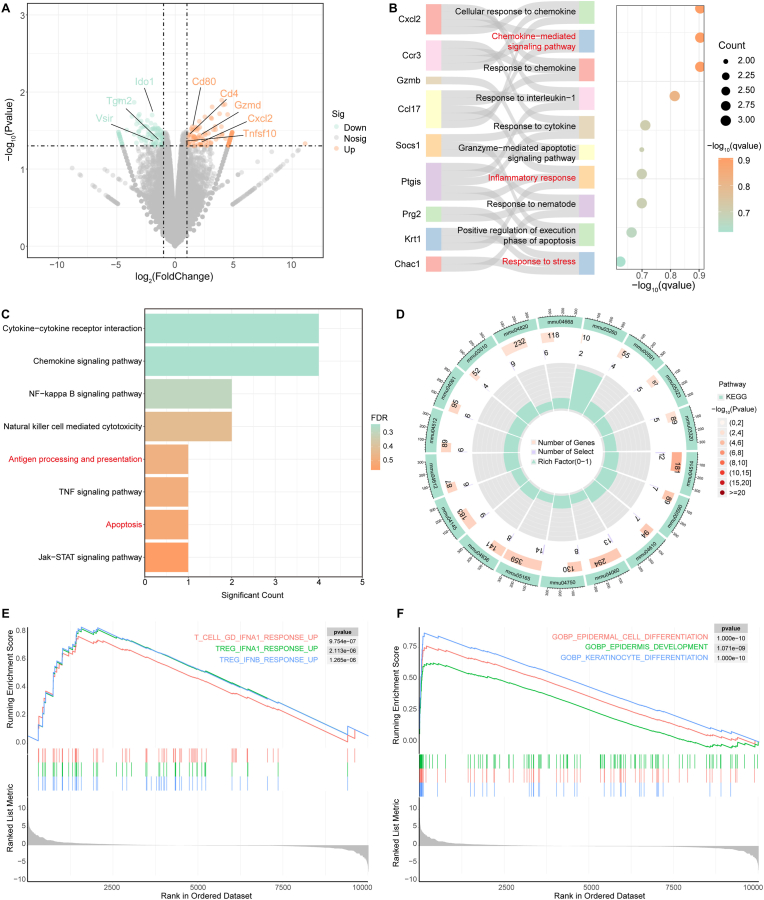
Biological mechanism of BCOP in anti-tumor therapy. (A) Volcano plot of DEGs (light green: downregulated genes; orange: upregulated genes). (B) GO enrichment analysis. (C) KEGG pathway enrichment. (D) Correlation analysis between DEGs and KEGG pathways. GSEA enrichment plots for DEGs in (E) immunology and (F) biological processes. (For interpretation of the references to color in this figure legend, the reader is referred to the Web version of this article.)

Building on the significant in vivo anti-tumor efficacy of BCOP, we further elucidated its underlying biological mechanisms through transcriptome sequencing (RNA-seq). Differentially expressed genes (DEGs) were screened using thresholds of |Log_2_FC| ≥ 1 and P < 0.05. BCOP + US treatment significantly altered the expression of 338 genes, with 156 upregulated and 182 downregulated (Fig. S16). The volcano plot revealed functional clustering among key DEGs: pro-apoptotic genes (Tnfsf10, Gzmd) and immune activation genes (CD80, CD4, Cxcl2) were significantly upregulated, while immunosuppressive genes (Vsir, Ido1, Tgm2) showed a downregulation trend (Fig. 6A). Tnfsf10, a key pro-apoptotic factor, binds to death receptors DR4/DR5 to initiate the tumor cell apoptosis program. CD80, a T-cell co-stimulatory molecule, mediates T-cell proliferation and cytokine secretion, potentiating the anti-tumor immune response [48]. CD4 participates in MHC-II complex recognition and T-cell activation, promoting cytokine production (e.g., IFN-γ) and synergistically regulating immune responses [49]. Based on the protein-protein interaction (PPI) network constructed from DEGs, this study further validated, at the protein functional collaboration level, the synergistic effects between the aforementioned apoptosis regulators (e.g., Tnfsf10) and immune activation molecules (e.g., CD80, CD4), thus linking transcriptomic alterations to the functional protein regulatory network (Fig. S17). These molecular mechanisms validate the phenotypic findings from flow cytometry and immunofluorescence staining, forming a closed evidence loop. Conversely, downregulation of the immunosuppressive factor Vsir alleviates its inhibition on T-cell activation and cytokine secretion, breaking the immune escape barrier within the tumor microenvironment. This expression profile corroborates the earlier flow cytometry and immunofluorescence results, revealing a trend of immune microenvironment reprogramming.

Gene Ontology (GO) enrichment analysis showed that DEGs were primarily concentrated in three core processes: oxidative stress response, regulation of programmed cell death, and coordinated recruitment of immune cells (Fig. 6B). The stress response pathway, activated by ROS, triggers tumor apoptosis and enhances immunogenicity through nodes like p53. Activation of the inflammatory response pathway not only directly kills tumor cells via ROS but also releases cytokines to initiate adaptive immunity. Activation of the chemokine-mediated signaling pathway recruits T cells, macrophages, and DCs to migrate into the tumor microenvironment, enhancing immune infiltration. A Sankey diagram further visualized the associations between DEGs and these key GO pathways. Kyoto Encyclopedia of Genes and Genomes (KEGG) pathway analysis confirmed significant activation of the apoptosis pathway and immune-related pathways such as antigen processing and presentation (Fig. 6C). A circular plot quantified the enrichment level of these pathways (Fig. 6D). Gene Set Enrichment Analysis (GSEA) revealed, at a systems level, that the therapy drives the anti-tumor effect through immune activation (e.g., T cell differentiation) and cellular fate reprogramming (e.g., differentiation regulation) (Fig. 6E and F).

Thus, BCOP-mediated sonodynamic-chemodynamic synergistically activated immunotherapy not only kills tumor cells by directly inducing apoptosis but also achieves immune-mediated destruction by reshaping the immune microenvironment, triggering ICD, and eliciting a systemic immune response, thereby realizing the dual anti-tumor effect of “tumor killing plus immune activation”.

## 3 Conclusion

In this study, we developed a bismuth-based heterojunction sonosensitizer, BiF_3_:Ce-BiOI-PEG (BCOP), via ion exchange, leveraging band engineering and interfacial engineering strategies. This synergistic engineering approach optimized the band structure through rare-earth Ce^3+^ doping and I^−^ exchange, forming a type-II heterojunction with a built-in electric field. Under US excitation, this structure significantly enhanced carrier separation efficiency, thereby substantially improving ROS generation efficacy. Simultaneously, BCOP exhibits intelligent TME-responsive properties, including a Ce^3+^-mediated Fenton-like reaction and dual GSH depletion pathways. These mechanisms synergistically weaken the antioxidant capacity of the TME. The resulting synergistic cycle of ROS burst and compromised antioxidant defense effectively induces mitochondrial damage and apoptosis in tumor cells. More importantly, BCOP efficiently triggers ICD, ultimately activating a robust systemic anti-tumor immune response. Experimental results confirmed that BCOP demonstrates excellent tumor suppression efficacy both in vitro and in vivo, alongside favorable biodegradability and biocompatibility. This multifunctional heterojunction design strategy provides an approach for optimizing sonosensitizer performance and advancing the development of nanomaterials for multimodal tumor therapy.

## CRediT authorship contribution statement

**Xueyu Li:** Writing – original draft, Visualization, Investigation, Conceptualization. **Jun Du:** Investigation. **Qingxuan Meng:** Investigation. **Lejin Zhu:** Investigation. **Yuqing Miao:** Supervision, Resources. **Yuhao Li:** Writing – review & editing, Project administration, Funding acquisition, Conceptualization. **Qing Miao:** Writing – review & editing, Project administration, Funding acquisition.

## Declaration of competing interest

The authors declare that they have no known competing financial interests or personal relationships that could have appeared to influence the work reported in this paper.

## Data Availability

Data will be made available on request.
