# Supplementary material for: Band-interface cooperative engineering of bismuth-based heterojunctions for sonodynamic-chemodynamic synergistic breast cancer therapy
Source: Mater Today Bio. 2025 Sep 6;35:102296. doi: 10.1016/j.mtbio.2025.102296 (PMC13014039; doi:10.1016/j.mtbio.2025.102296)
Supplement: Multimedia component 1 [file mmc1.docx]

**Supporting Information**

Band-Interface Cooperative Engineering of Bismuth-Based Heterojunctions for Sonodynamic-Chemodynamic Synergistic Breast Cancer Therapy

Xueyu Li^1,2^, Jun Du^2^, Qingxuan Meng^2^, Lejin Zhu^2^, Yuqing Miao^2^, Yuhao Li^1,2^*, Qing Miao^1^*

1. Department of Anesthesiology, Jiading District Central Hospital Affiliated Shanghai University of Medicine and Health Sciences, Shanghai University of Medicine and Health Sciences, Shanghai 201318, China

2. School of Materials and Chemistry, Institute of Bismuth Science, Shanghai Collaborative Innovation Center of Energy Therapy for Tumors, University of Shanghai for Science and Technology, Shanghai 200093, China

Corresponding author:

Yuhao Li, E-mail: [yhli@usst.edu.cn](mailto:yhli@usst.edu.cn)

Qing Miao, E-mail: [miaoqmz@163.com](mailto:miaoqmz@163.com)

**1. Materials**

All chemicals were purchased from commercial sources and used without further purification. Bi(NO_3_)_3_·5H_2_O (99.9%), Ce(NO_3_) _3_·6H_2_O (99.9%), Bi_2_O_3_ (99.9%), NH_4_F (99.9%), and KI (99%) were obtained from Adamas (China). Ethylene glycol (EG, 99.0%) and glycerol (99.0%) were acquired from Shanghai Chemical Reagent Company (China).

**2. Synthesis of BiF_3_:Ce (BC) Nanoparticles**

Bi(NO_3_)_3_·5H_2_O (0.08 mM, 388.0 mg) and Ce(NO_3_)_3_·6H_2_O (0.02 mM, 86.8 mg) were dissolved in EG (10 mL). After complete dissolution, an EG solution (25 mL) containing NH_4_F (0.3 mM) was added. The reaction was terminated after vigorous stirring for 60 s. The product was collected by centrifugation, washed three times with H_2_O, dispersed in H_2_O, and stored at 4 ℃.

**3. Synthesis of BiF_3_:Ce-BiOI (BCO)**

As-synthesized BC (2 mg) was dispersed in a mixed solution of H_2_O (2 mL) and glycerol (5 mL). This dispersion was then added to an aqueous solution (5 mL) containing KI (0.5 mM). After 3 h, the reaction was stopped, and the product was collected by centrifugation. The product was washed three times with H_2_O, dispersed in H_2_O, and stored at 4 ℃.

**4. Synthesis of BiF_3_:Ce-BiOI-PEG (BCOP)**

BCO (2 mg) was dispersed in anhydrous ethanol (2 mL), followed by the addition of PEG (6 mg). The mixture was stirred in the dark for 24 h. After stirring, the product was collected by centrifugation. The final product was dispersed in PBS and stored at 4°C.

**5. Photoelectrochemical Performance Testing**

The current response of BCOP was measured using an electrochemical workstation (CHI760E, China). A working electrode was fabricated by coating BCOP onto fluorine-doped tin oxide (FTO) glass. A three-electrode system was assembled using this working electrode, an Ag/AgCl reference electrode, and a platinum foil counter electrode. The system was placed in a quartz electrolytic cell containing phosphate-buffered saline (PBS, pH 7.4). The current response under ultrasound (US) irradiation (WED-100, China, 1 MHz, 0.7 W cm^−2^) was recorded. EIS was performed in an electrolyte containing [Fe(CN)_6_]^3−^ (5 mM) and KCl (0.1 M) with an amplitude of 0.005 V over a frequency range of 100 kHz to 0.1 Hz.

**6. In Vitro Detection of Reactive Oxygen Species (ROS)**

The ROS probe DPBF was used to detect ROS generated by BCOP under ultrasound irradiation. A mixture of BCOP and DPBF (80 µM, 2 mL) was subjected to ultrasound irradiation (1 MHz, 0.7 W cm^−2^) for varying durations. Absorbance changes were detected using an absorption spectrophotometer. The characteristic absorption peak of DPBF is at 421 nm. Absorbance at 421 nm for different time points was normalized using the formula: A = A_t min_ / A_0 min_ × 100%, where A_t min_ and A_0 min_ are the absorbances at 421 nm before and after t minutes of ultrasound irradiation, respectively.

**7. Detection of Hydroxyl Radical Generation for Chemodynamic Therapy**

TMB (3,3’,5,5’-tetramethylbenzidine) was employed as an indicator for •OH detection. BCOP (0, 25, 50, 75, 100, 150 µg mL^−1^), H_2_O_2_ (10 mM), and TMB (0.8 mM) were mixed in 3 mL of PBS at different pH values (6.5 or 5.5). After incubation at room temperature for 8 min, the absorbance of ox-TMB at 655 nm and 895 nm was measured to evaluate the chemodynamic performance.

**8. Electron Spin Resonance Detection of ROS**

DMPO and TEMP were used as spin traps to detect ROS generation. BCOP (100 µg mL^−1^) was mixed with DMPO or TEMP. After ultrasound irradiation (1 MHz, 0.7 W cm^−2^, 3 min), ESR signals were detected using an electron paramagnetic resonance spectrometer.

**9. TME-Responsive Degradation Behavior of BCOP**

Degradation experiments were performed on BCOP (100 µg mL^−1^) in four different buffer systems: pH 7.4/GSH, pH 7.4/GSH/H_2_O_2_, pH 5.5/GSH, pH 5.5/GSH/H_2_O_2_ (GSH 10 mM, H_2_O_2_ 10 mM). BCOP was mixed with the respective buffers and incubated in a thermostatic shaker (37 °C, 120 rpm). Absorbance at 348 nm was measured at various time points to monitor GSH consumption. Morphological evolution of BCOP in different buffer systems over time (1, 3, 6, 12, 24 h) was observed by TEM.

**10. In Vitro Glutathione Depletion Assay**

The DTNB (5,5’-dithiobis(2-nitrobenzoic acid)) reaction method was utilized to quantify GSH depletion. BCOP (100 µg mL^−1^) was incubated with buffers as described previously. At different time points, the mixture was centrifuged (13500 rpm, 10 min) to remove undegraded nanoparticles. The supernatant was mixed with DTNB (3 mg mL^−1^) and transferred to a 96-well plate. Absorbance at 412 nm was measured using a microplate reader.

**11. Cell Culture**

Mouse breast cancer cells (4T1) were purchased from the Shanghai Institute of Biological Sciences (Chinese Academy of Sciences, China). 4T1 cells were cultured in RPMI-1640 medium (Adamas, China) supplemented with 10% fetal bovine serum (FBS) in an incubator (37 °C, 5% CO_2_, ThermoFisher, USA).

**12. Cell Viability Assessment**

Cell viability was assessed using the Cell Counting Kit-8 (CCK-8) assay. 4T1 cells were seeded in 96-well plates at a density of 1×10^3^ cells/well. After 12 h, cells were treated under different conditions and incubated for 4 h. Cells were then washed with fresh RPMI-1640 medium and subjected to ultrasound irradiation (1.0 MHz, 0.7 W cm^−2^, 7 min). After further incubation for 20 h, cells were washed with PBS, and CCK-8 reagent was added for 40 min incubation. Absorbance at 450 nm was measured using a microplate reader. Cell viability was calculated as: Cell viability (%) = (Absorbance of treated group) / (Average absorbance of control group) × 100%.

**13. Detection of Intracellular ROS Levels**

The ROS probe 2’,7’-dichlorodihydrofluorescein diacetate (DCFH-DA, Beyotime, China) was used to detect intracellular ROS levels. 4T1 cells were seeded in 12-well plates at a density of 1×10^4^ cells/well and cultured for 24 h. After incubation with BCOP (100 µg mL^−1^), cells were treated with ultrasound irradiation (1.0 MHz, 0.7 W cm^−2^, 7 min). Cells were then incubated with DCFH-DA in the dark for 30 min according to the manufacturer’s instructions and washed with PBS. Intracellular green fluorescence was observed using a fluorescence microscope (Olympus IX73, Japan).

**14. Live/Dead Cell Staining**

Live and dead cells were stained using Calcein-AM and propidium iodide (PI) (Sigma-Aldrich, USA), respectively. 4T1 cells were seeded in 24-well plates and cultured for 12 h. Cells were then treated with PBS/RPMI-1640 solution containing BCOP for 4 h, and relevant groups were exposed to ultrasound irradiation (1.0 MHz, 0.7 W cm^−2^, 7 min). After further incubation for 20 h, cells were incubated with Calcein-AM/PI solution for 20 min. After washing three times with PBS, green fluorescence (Calcein-AM) and red fluorescence (PI) were captured and imaged using a fluorescence microscope.

**15. Mitochondrial Membrane Potential Staining**

Changes in mitochondrial membrane potential of 4T1 cells were evaluated using the JC-1 Mitochondrial Membrane Potential Assay Kit (Beyotime, China). 4T1 cells were seeded in 12-well plates at a density of 1×10^4^ cells/well and cultured for 24 h. Groups were incubated with BCOP (100 µg mL^−1^) for 24 h, followed by group-specific ultrasound irradiation (1.0 MHz, 0.7 W cm^−2^, 7 min). After 12 h, cells were washed twice with PBS, incubated with JC-1 staining working solution in the dark for 30 min, and imaged using a fluorescence microscope. JC-1 monomers appear as green fluorescence, while JC-1 aggregates appear as red fluorescence.

**16. Apoptosis Detection**

Cell apoptosis was evaluated using an Annexin V-FITC/PI Apoptosis Detection Kit. 4T1 cells were seeded in 6-well plates at a density of 3×10^4^ cells/well. After incubation with BCOP (100 µg mL^−1^) and ultrasound treatment (1.0 MHz, 0.7 W cm^−2^, 7 min), cells were washed, trypsinized, centrifuged, and collected. Cells were processed according to the kit instructions and incubated with Annexin V-FITC and PI in the dark. Finally, cell apoptosis was detected by flow cytometry.

**17. CRT and HMGB1 Detection**

Calreticulin (CRT) and high-mobility group box 1 (HMGB1) levels were assessed by immunofluorescence staining. Cells were seeded in 12-well plates at a density of 1×10^4^ cells/well and cultured for 24 h. Groups were incubated with BCOP (100 µg mL^−1^) for 24 h, followed by group-specific ultrasound irradiation (1.0 MHz, 0.7 W cm^−2^, 7 min). CRT staining was performed at 6 h post-treatment; HMGB1 staining was performed at 12 h post-treatment. Cells were washed with PBS, fixed with 4% paraformaldehyde for 20 min, and permeabilized with 0.1% Triton X-100 (Servicebio, China) for 10 min. After blocking with 10% bovine serum albumin (BSA, Servicebio, China), cells were incubated with primary antibodies against CRT and HMGB1 (Servicebio, China) at room temperature for 2 h, followed by incubation with Alexa Fluor 488-conjugated secondary antibody for 1 h. Finally, nuclei were counterstained with DAPI for 10 min, and samples were observed under a fluorescence microscope.

**18. ATP Release**

Cells were seeded in 12-well plates at a density of 1×10^4^ cells/well and cultured for 24 h. Groups were incubated with BCOP (100 µg mL^−1^) for 24 h, followed by group-specific ultrasound irradiation (1.0 MHz, 0.7 W cm^−2^, 7 min). After 12 h, cell culture supernatants were collected. ATP levels in the supernatants were measured using an ATP Assay Kit (Beyotime, China) according to the manufacturer’s instructions. Quantification was performed using a multimode microplate reader (Tecan Spark, Switzerland).

**19. Tumor Model**

A 4T1 tumor model was established in 5-week-old female Balb/c mice. Mice were purchased from the Laboratory Animal Management Department of the Shanghai Institute of Planned Parenthood Research. All animal experiments were approved by the Shanghai Zhibei Biotechnology Co., Ltd. Animal Care and Use Committee (IRB-AF68). A 4T1 cell suspension (100 μL, 2×10^6^ cells mL^−1^) was injected subcutaneously into the right posterior armpit as the primary tumor. Approximately 4 days later, a distant tumor was established in the left posterior armpit using the same method.

**20. Biodistribution**

BCOP was labeled with the near-infrared fluorescent dye IR-780 for in vivo fluorescence imaging. IR-780 (0.5 mg) was thoroughly mixed with BCOP (1 mg mL^−1^, 2 mL) and stirred in the dark for 24 h. Subsequently, BCOP-IR780 was washed with PBS and centrifuged. Before use, BCOP-IR780 was dispersed in PBS. BCOP-IR780 (1 mg mL^−1^, 100 μL) was injected intravenously via the tail vein. Fluorescence images of mice were acquired at different time points (1, 3, 6, 12, 24 h) using a small animal in vivo imaging system (PerkinElmer IVIS Lumina III, USA, λ_ex_ = 630 nm, λ_em_ = 800 nm). Finally, mice were sacrificed, and major organs and tumors were harvested for fluorescence imaging.

**21. Hemolysis Assay**

Mouse red blood cells (RBCs) were collected to evaluate the hemolytic activity of BCOP. Blood (1 mL) obtained via orbital bleeding was mixed with PBS (2 mL) and centrifuged (1200 rpm, 5 min). After discarding the supernatant, RBCs were resuspended in PBS until the supernatant was colorless. RBCs were then dispersed in PBS (3 mL). RBC suspension (100 μL) was incubated with deionized water (positive control), PBS (negative control), or different concentrations of BCOP (25, 50, 75, 100, 150 µg mL^−1^). After incubation for 5 h, solutions were centrifuged (13500 rpm, 10 min), and the absorbance of the supernatant at 570 nm was recorded. The hemolysis rate was calculated as: Hemolysis (%) = (A_sample_ − A_PBS_) / (A_H2O_ − A_PBS_) × 100%.

**22. Hematological and Organ Damage Analysis**

Mice were injected intravenously with BCOP (1 mg mL^−1^, 100 μL) or PBS (100 μL, control). Blood samples were collected on day 21 for hematological analysis. Heart, liver, spleen, lungs, and kidneys were fixed in formalin solution, embedded in paraffin, sectioned, and stained with hematoxylin and eosin (H&E).

**23. Tumor Therapy**

The tumor-bearing mice were randomly divided into 4 groups (n = 5): (1) Control, (2) US (1.0 MHz, 0.7 W cm^−2^, 7 min), (3) BCOP (1 mg mL^−1^, 100 μL), and (4) BCOP+US (BCOP+US). Twelve hours after injection, the mice received US treatment. During the treatment period, the mice underwent two treatments (at day 0.5 and day 3.5). Tumor volume and body weight were recorded every two days. The tumor volume was calculated using the formula: tumor volume = (length × width^2^) / 2. Fourteen days later, the mice in each group were euthanized, and the tumors were excised, photographed, and weighed. The tumor tissues from each group were prepared into sections for immunohistochemical staining and histological analysis.

**24. Evaluation of Anti-Tumor Immune Response**

Fourteen days post-treatment, 4T1 tumor-bearing mice were sacrificed, spleens were collected and homogenized. The homogenate was filtered through a nylon mesh to obtain a single-cell suspension. The Live/Dead Fixable Violet Dead Cell Stain Kit was used to distinguish live/dead cells, followed by incubation with PBS containing anti-CD16/32 mAb to block Fc receptors. Subsequently, cells were surface-stained with fluorescently labeled CD45, CD3, CD4, and CD8 antibodies for 30 min. After staining, cells were fixed and permeabilized. Finally, cells were analyzed using a flow cytometer (BD FACSCanto II, USA), and data were processed using FlowJo software. All antibodies were purchased from Biolegend (USA).

**25. RNA Sequencing**

One day after different treatments, 4T1 tumor-bearing mice were sacrificed, and tumor tissues from the Control and BCOP+US groups were collected. High-throughput sequencing and data analysis were performed by Shanghai Gene Biotech Co., Ltd.

**26. Statistical Analysis**

Experimental data are presented as mean ± standard deviation (SD). A two-tailed, unpaired Student’s t-test was employed for comparisons between two groups, while one-way analysis of variance (ANOVA) was utilized for multiple-group comparisons. Statistical significance is denoted in figures as follows: * p < 0.05, ** p < 0.01, *** p < 0.001.


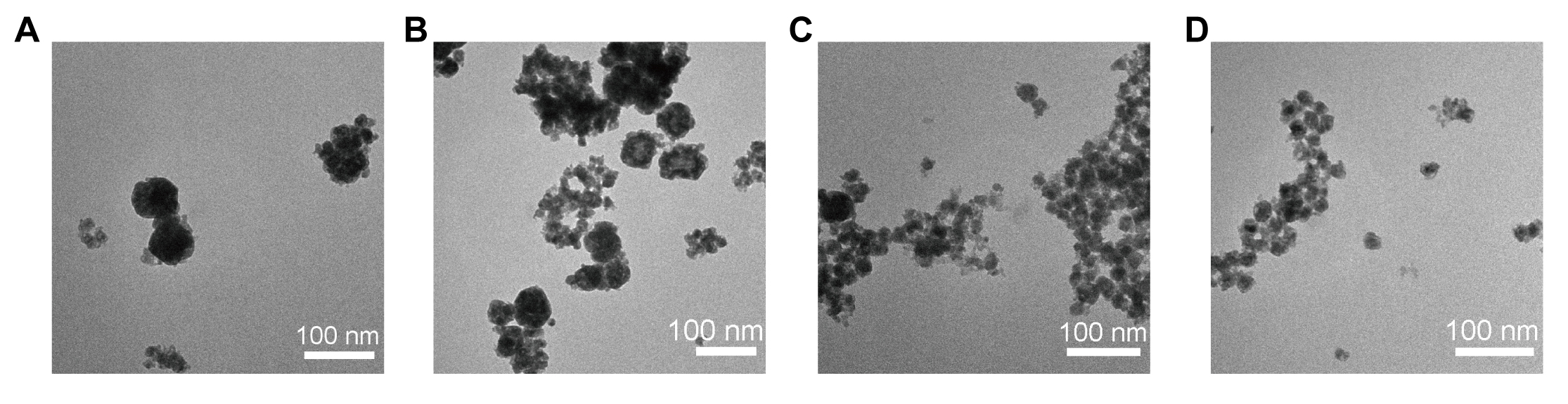


**Fig. S1.** TEM images of BiF_3_:Ce doped with different Ce^3^^+^ ratios: (A) 5%, (B) 10%, (C) 15%, (D) 20%.


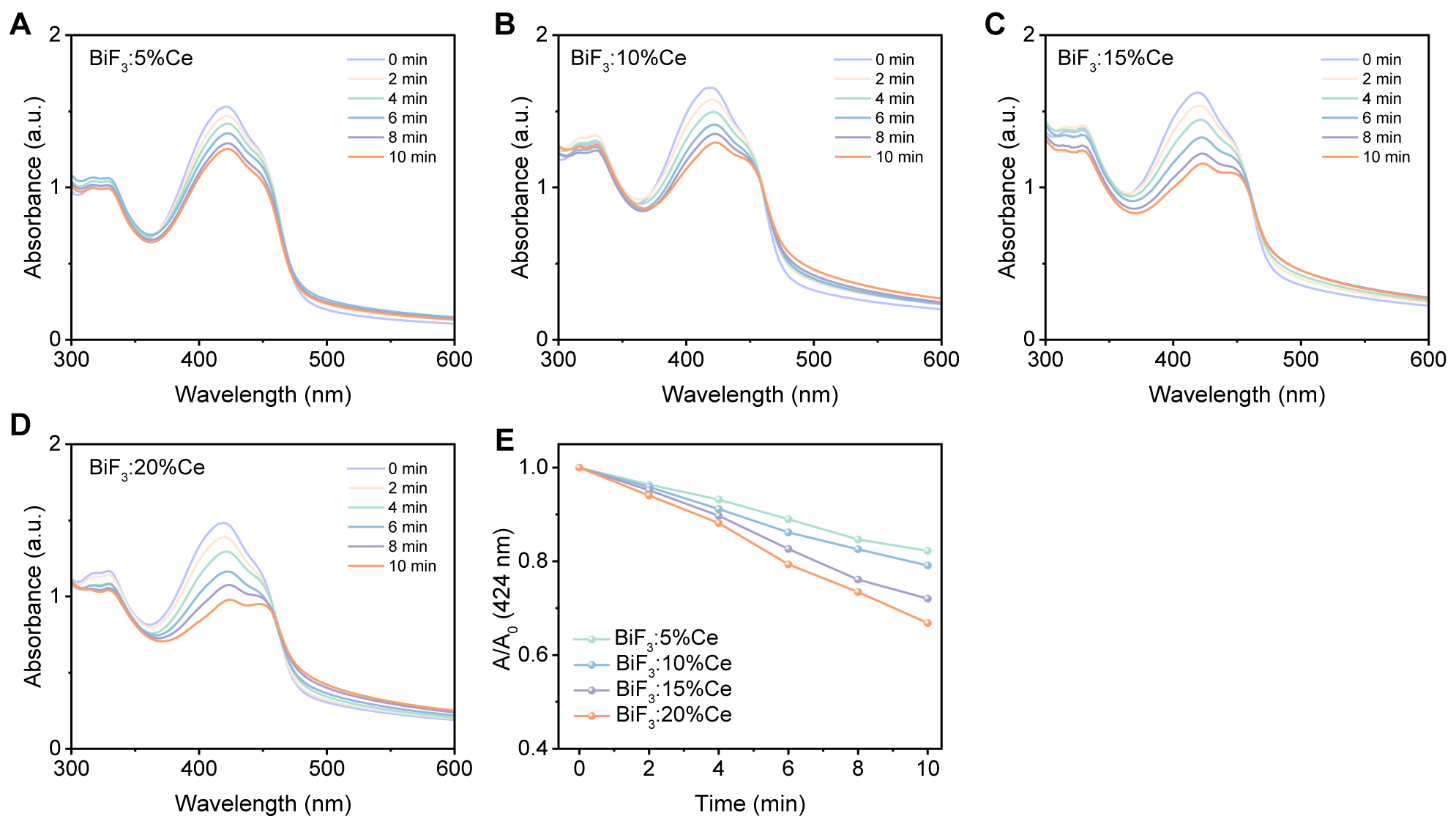


**Fig. S2.** Time-dependent absorption spectra of DPBF mixed with BiF_3_:Ce at different Ce^3+^ doping ratios: (A) 5%, (B) 10%, (C) 15%, (D) 20%, under US irradiation. (E) Absorbance changes of DPBF at 424 nm.


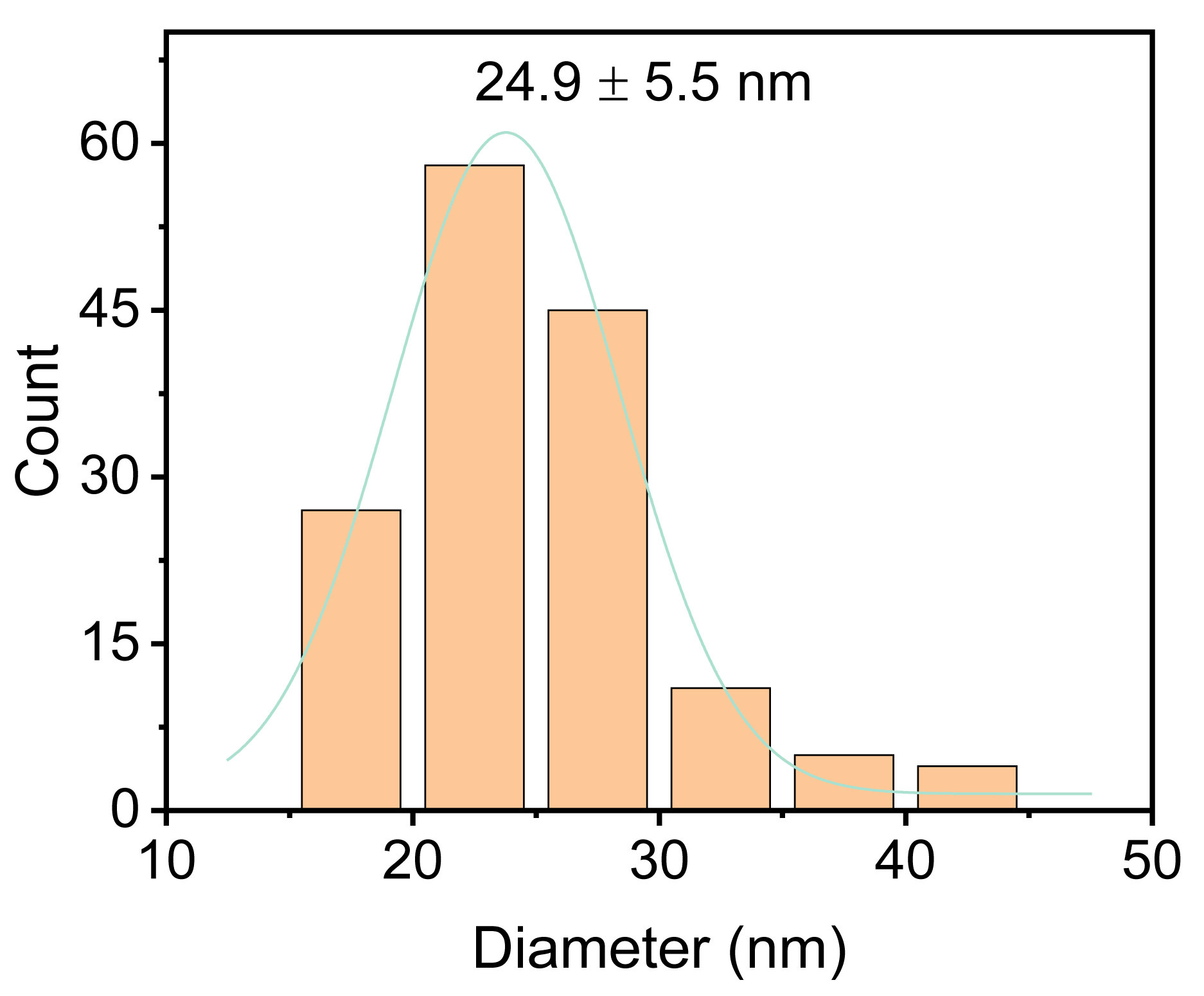


**Fig. S3.** Particle size distribution statistics of BCO.


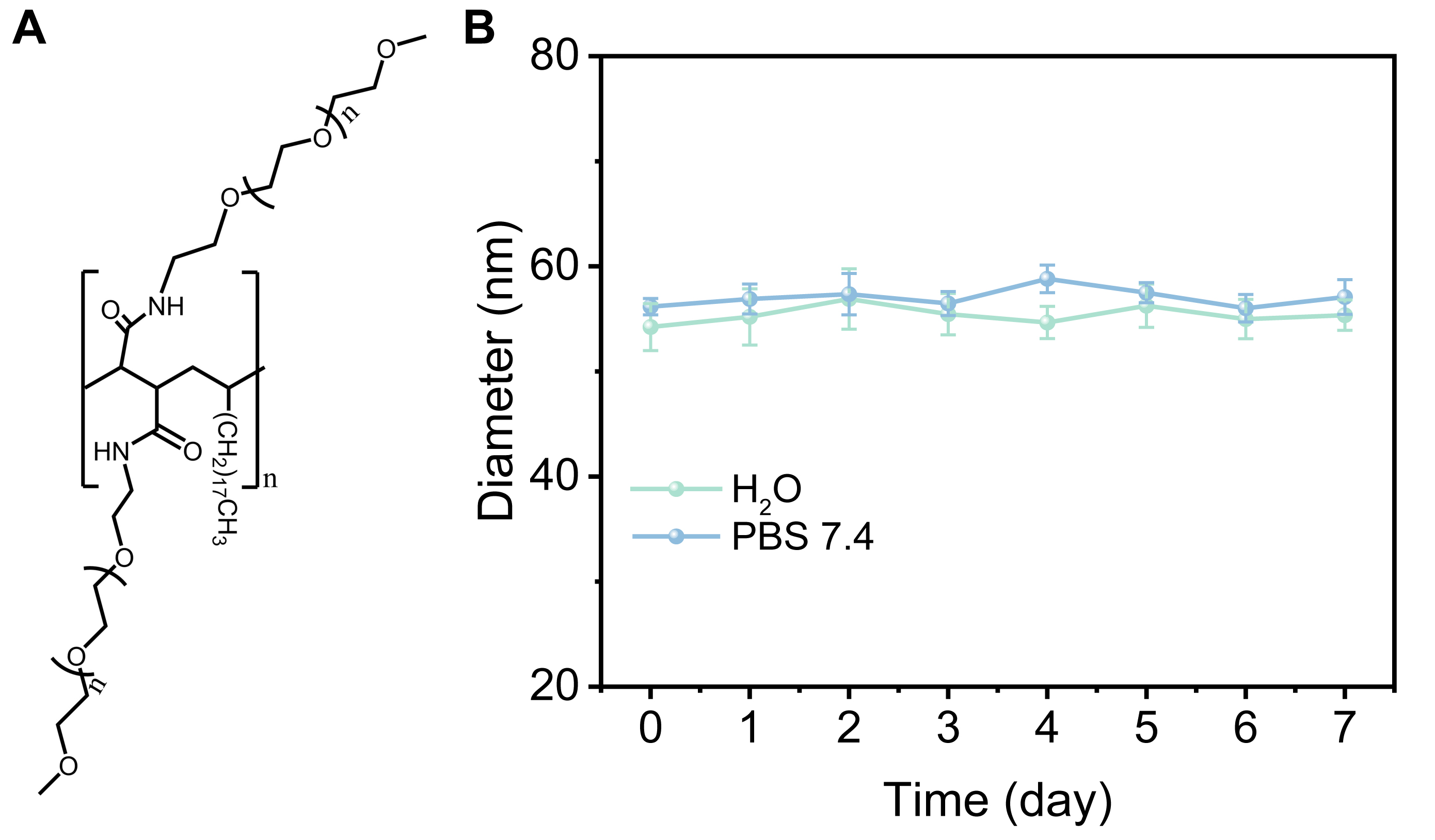


**Fig. S4.** (A) The structural formula of PEG. (B) Changes in DLS of BCOP in H_2_O and PBS (pH 7.4) over 7 days (Mean ± SD, n = 3).


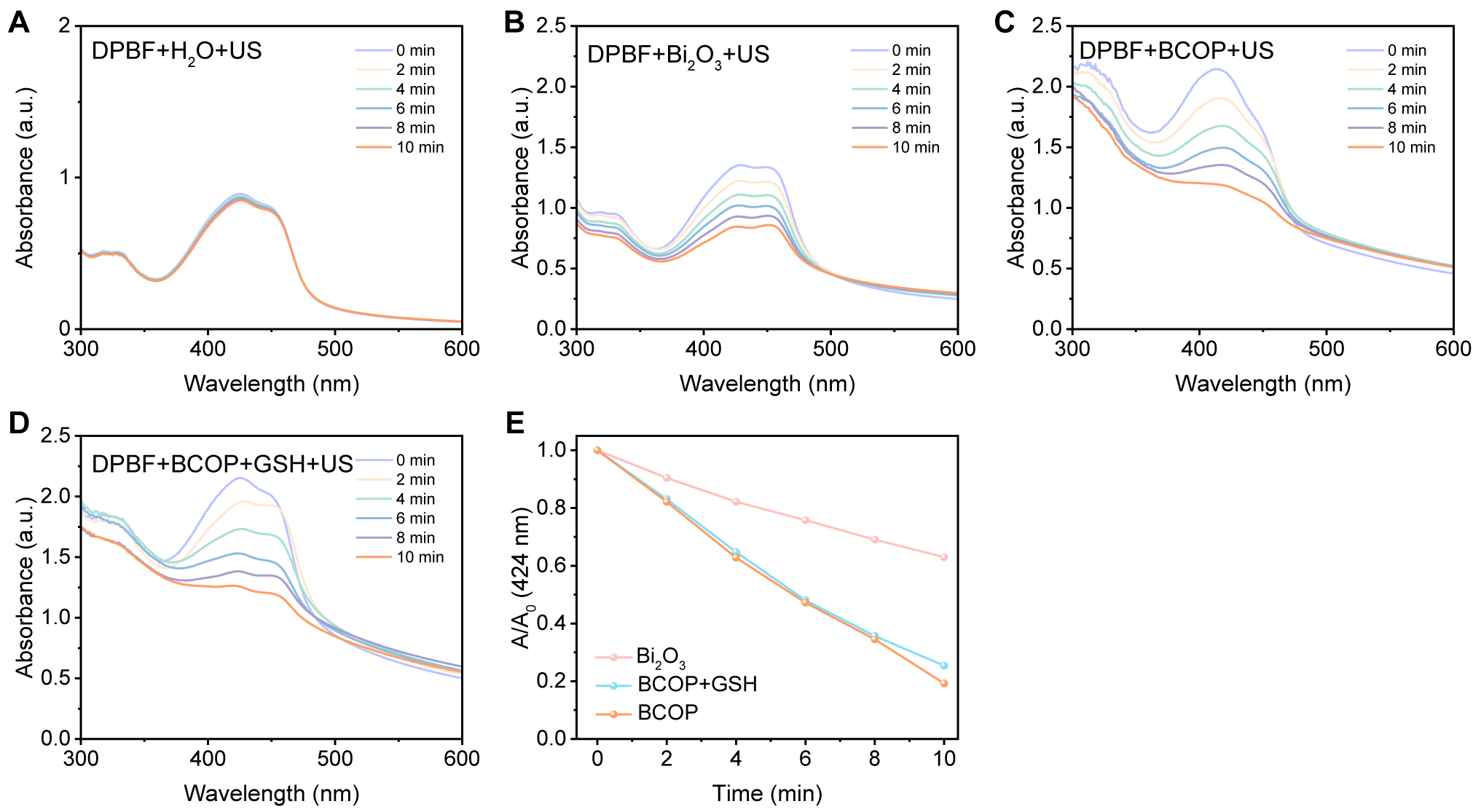


**Fig. S5.** Time-dependent absorption spectra of DPBF mixed with (A) H_2_O, (B) Bi_2_O_3_, (C) BCOP, and (D) BCOP + GSH under US irradiation. (E) Absorbance changes of DPBF at 424 nm.


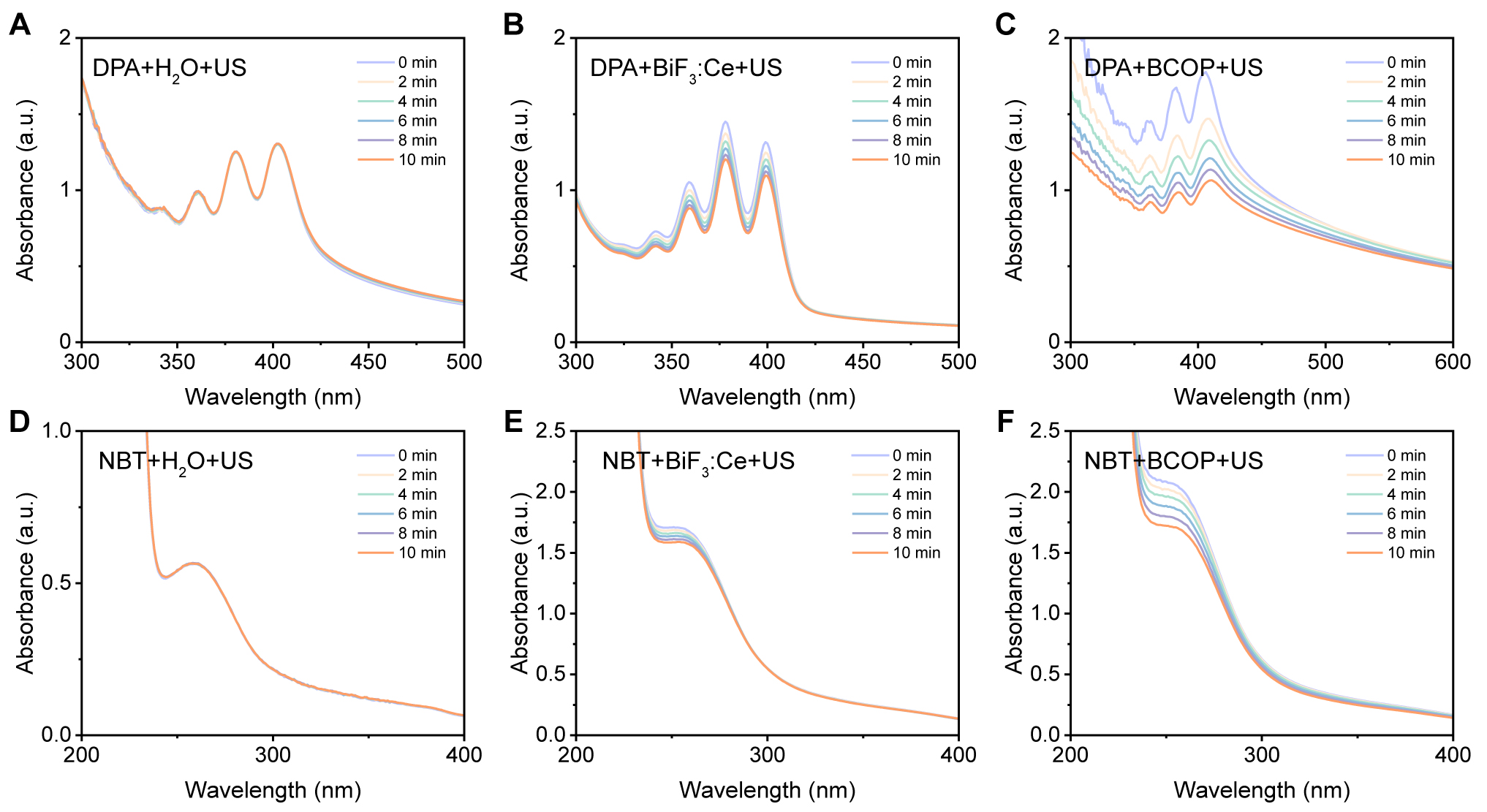


**Fig. S6.** Time-dependent absorption spectra of DPA mixed with (A) H_2_O, (B) BiF_3_:Ce, and (C) BCOP under US irradiation. Time-dependent absorption spectra of NBT mixed with (D) H_2_O, (E) BiF_3_:Ce, and (F) BCOP under US irradiation.


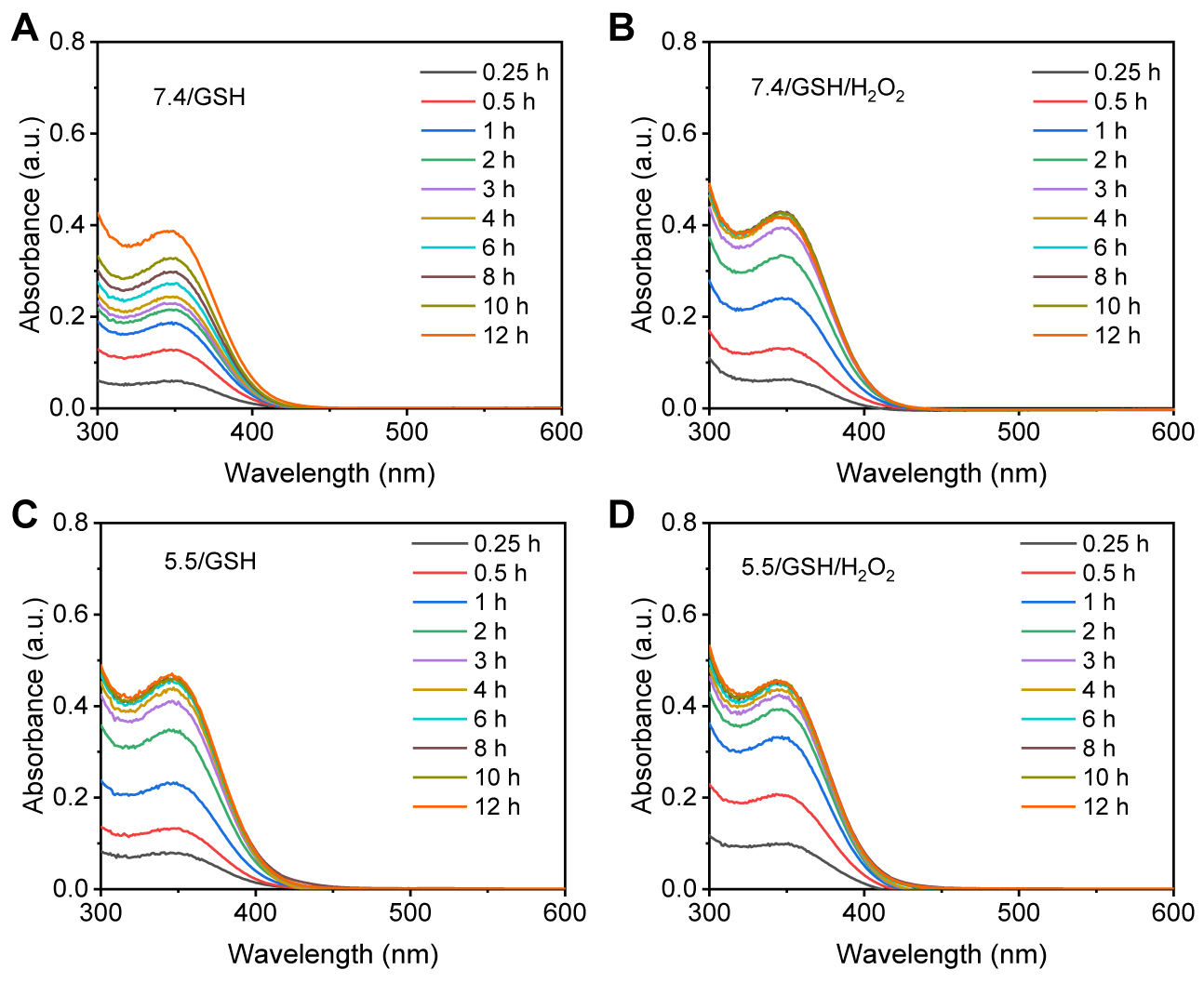


**Fig. S7.** Time-dependent absorbance changes of BCOP supernatants at 348 nm under conditions: (A) pH 7.4 + GSH, (B) pH 7.4 + GSH + H_2_O_2_, (C) pH 5.5 + GSH, (D) pH 5.5 + GSH + H_2_O_2_.


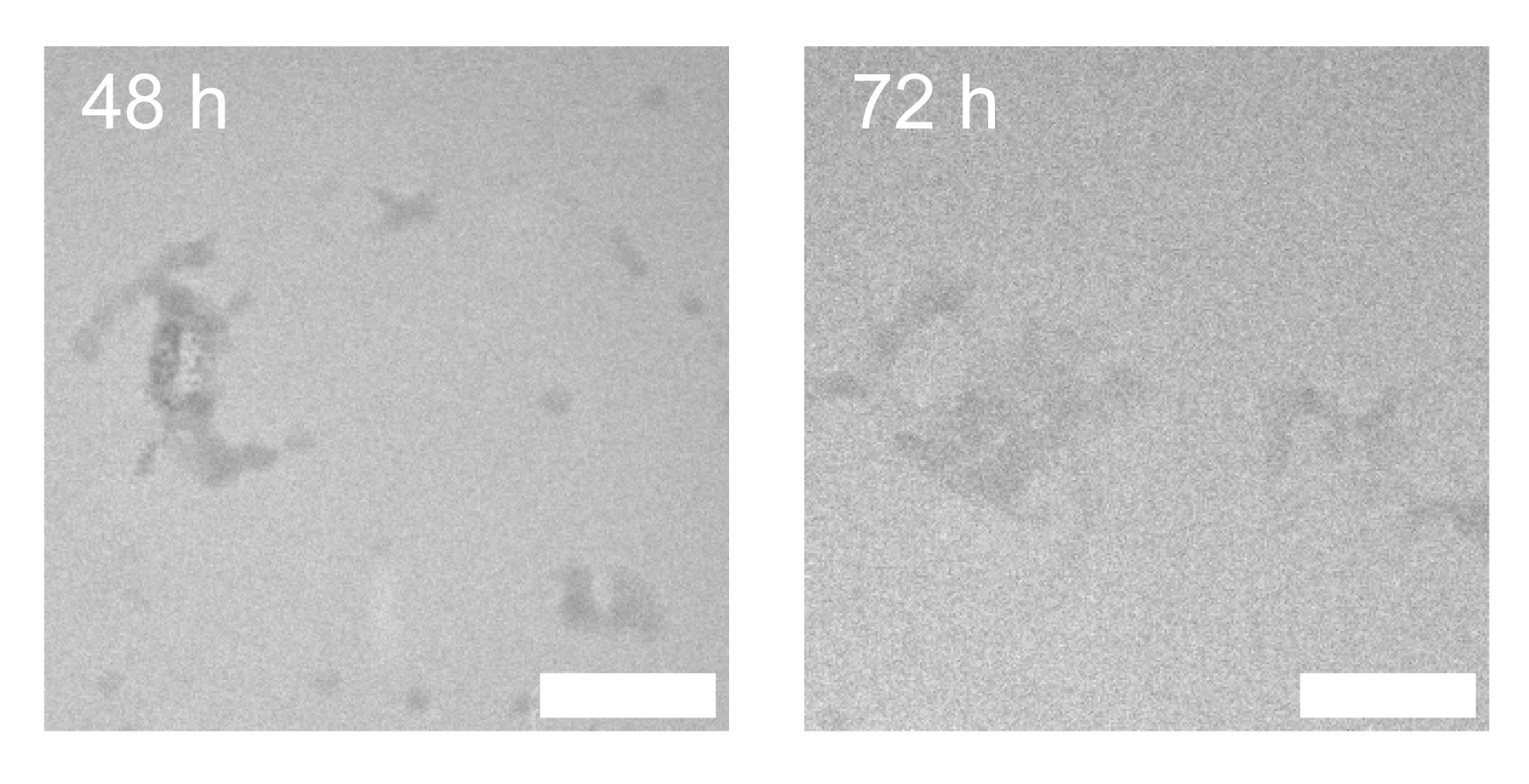


**Fig. S8.** TEM images of BCOP at 24 h and 48 h under pH 5.5, GSH, and H₂O₂ conditions (Scale bar: 200 nm).


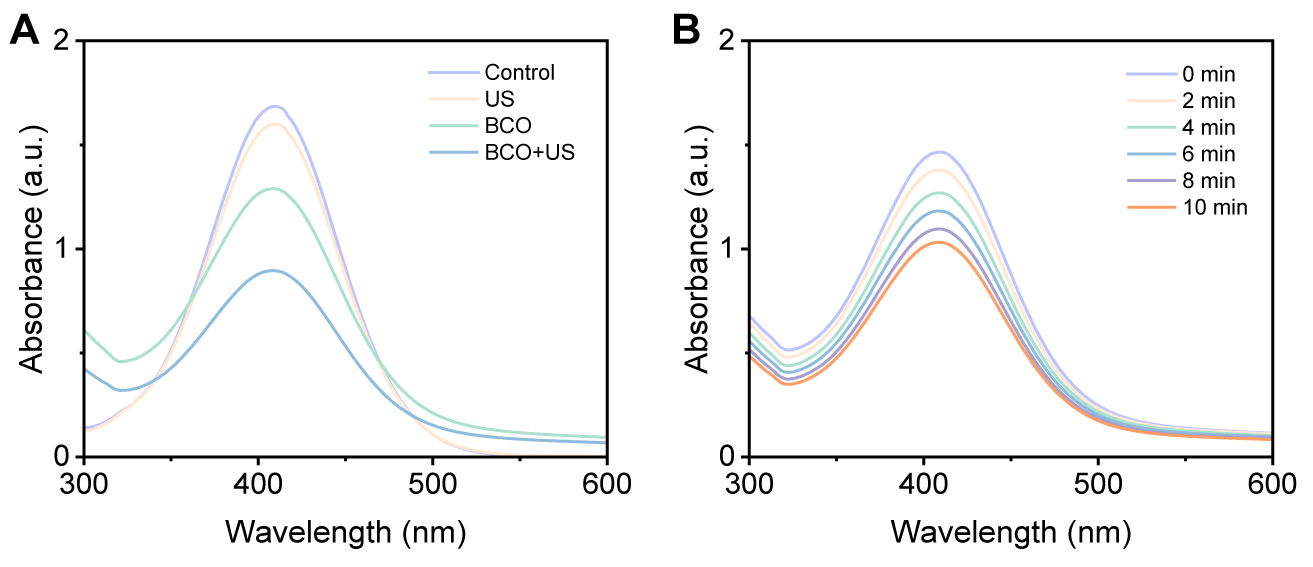


**Fig. S9.** (A) Absorption spectra of DTNB in GSH mixtures across different groups. (B) Time-dependent absorption spectra of DTNB in BCOP/GSH mixtures under US irradiation.


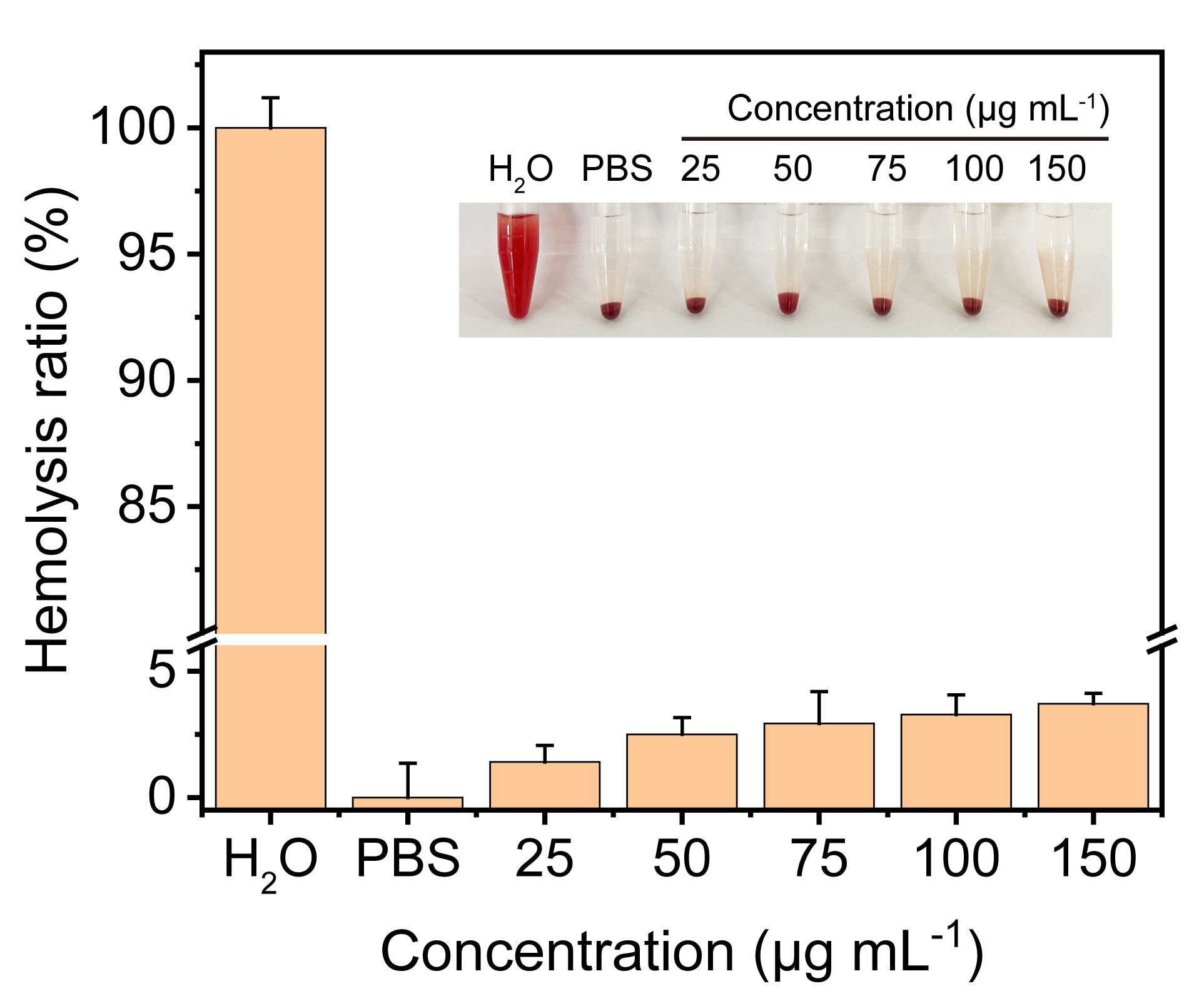


**Fig. S10.** Hemolysis analysis of BCOP at different concentrations; inset shows photographic evidence of hemolysis (Mean ± SD, n = 3).


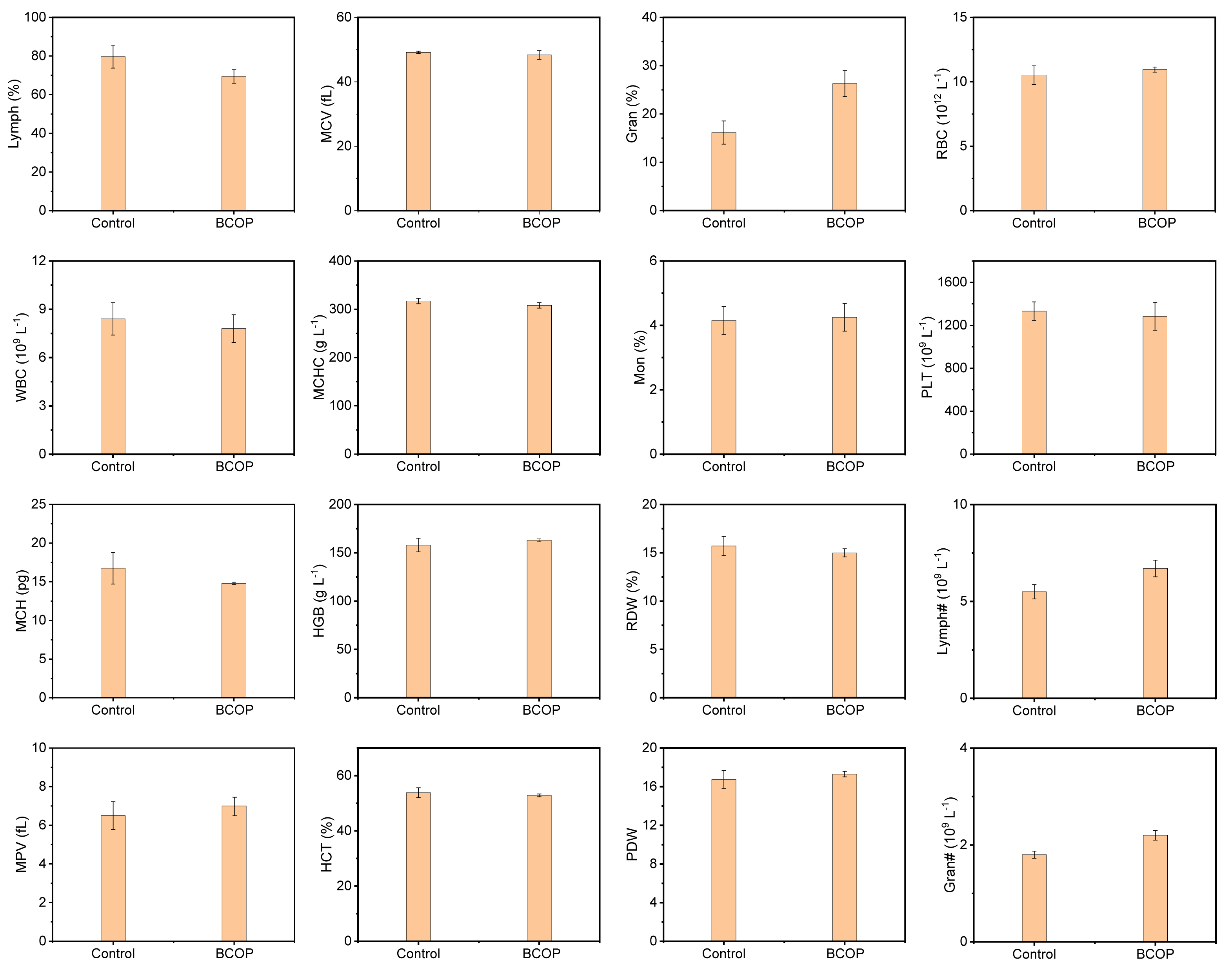


**Fig. S11.** Hematological analysis of mice 14 days post-injection with PBS (100 μL) or BCOP (1 mg mL^−1^) (Mean ± SD, n = 3).


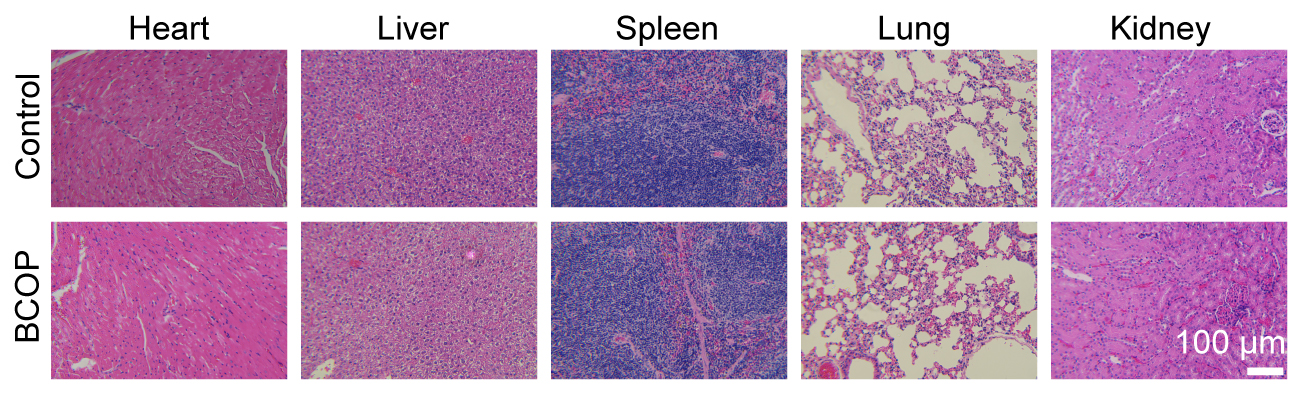


**Fig. S12.** H&E staining of major organs from mice 14 days post-injection with PBS (100 μL) or BCOP (1 mg mL^−1^).


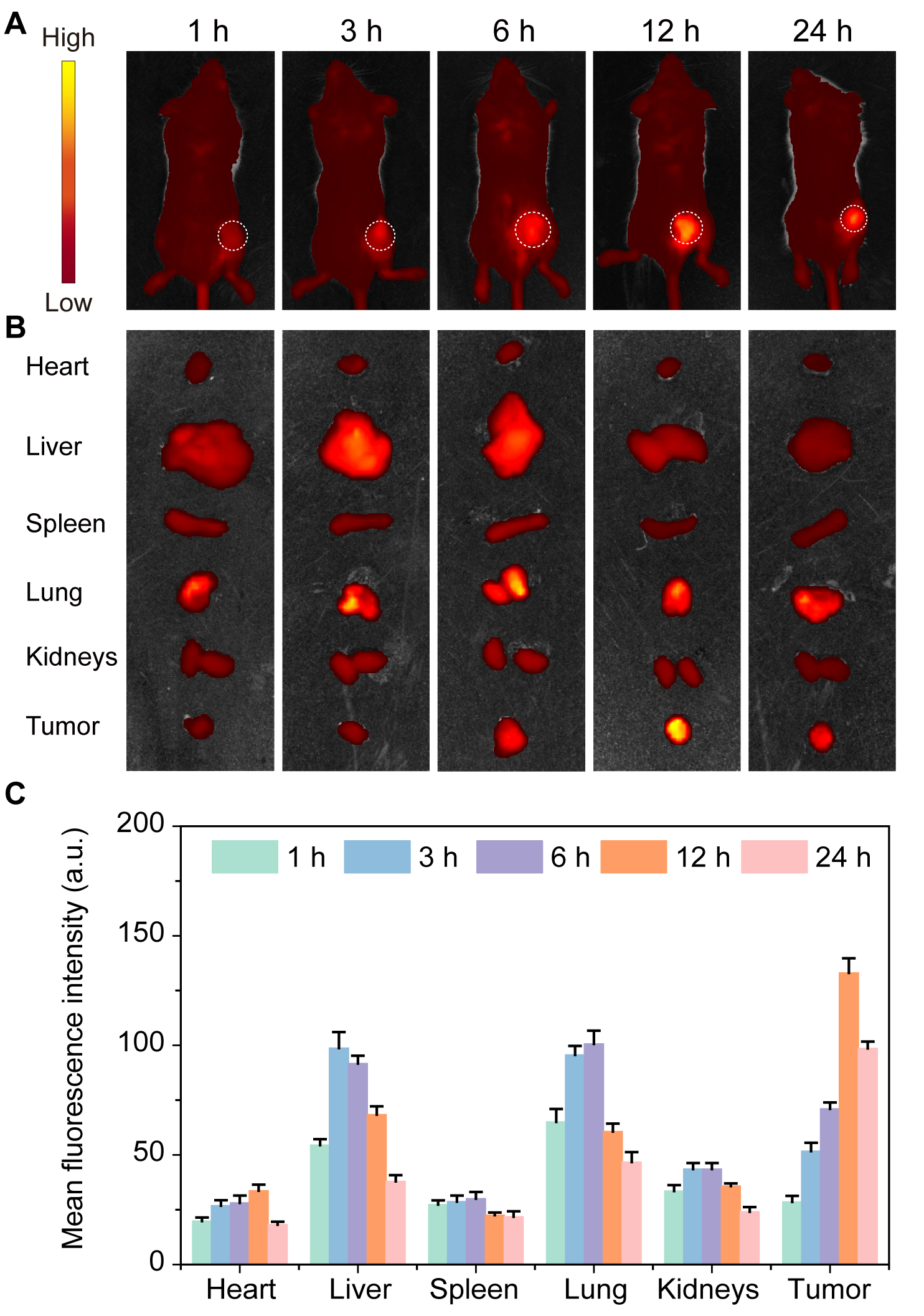


**Fig. S13.** (A) Fluorescence imaging of 4T1 tumor-bearing mice at different time points after intravenous injection of BCOP-IR780 (1 mg mL^−1^, 100 μL) via the tail vein. (B) Ex vivo imaging of major organs and tumors. (C) Corresponding quantitative fluorescence analysis (Mean ± SD, n = 3).


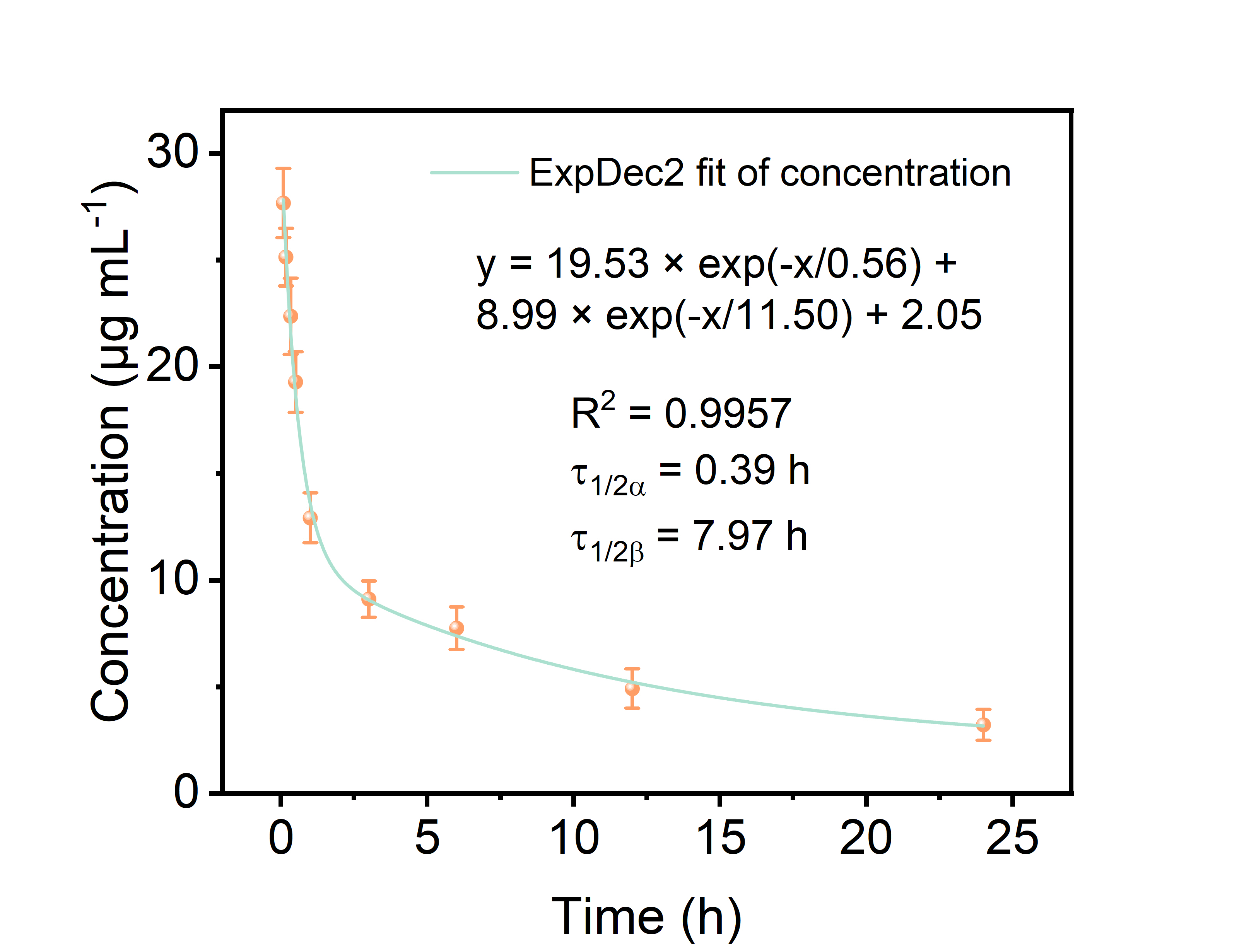


**Fig. S14.** Concentration-time curve of BCOP (1 mg mL^−1^, 100 μL) in blood following tail vein injection (Mean ± SD, n = 3).


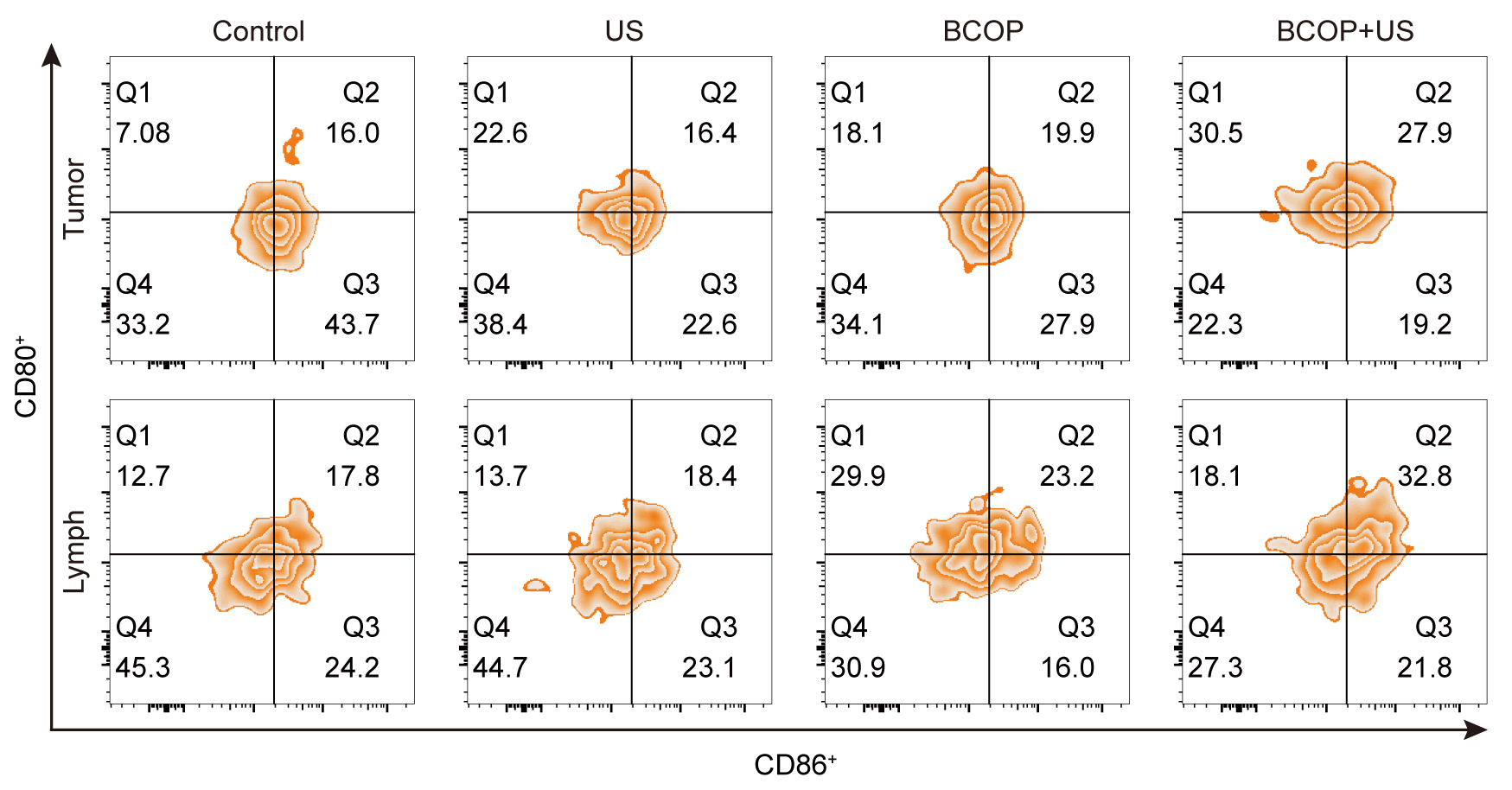


**Fig. S15.** (A) Flow cytometry analysis of DC maturation in tumors. (B) Flow cytometry analysis of DC maturation in lymphoid tissue.


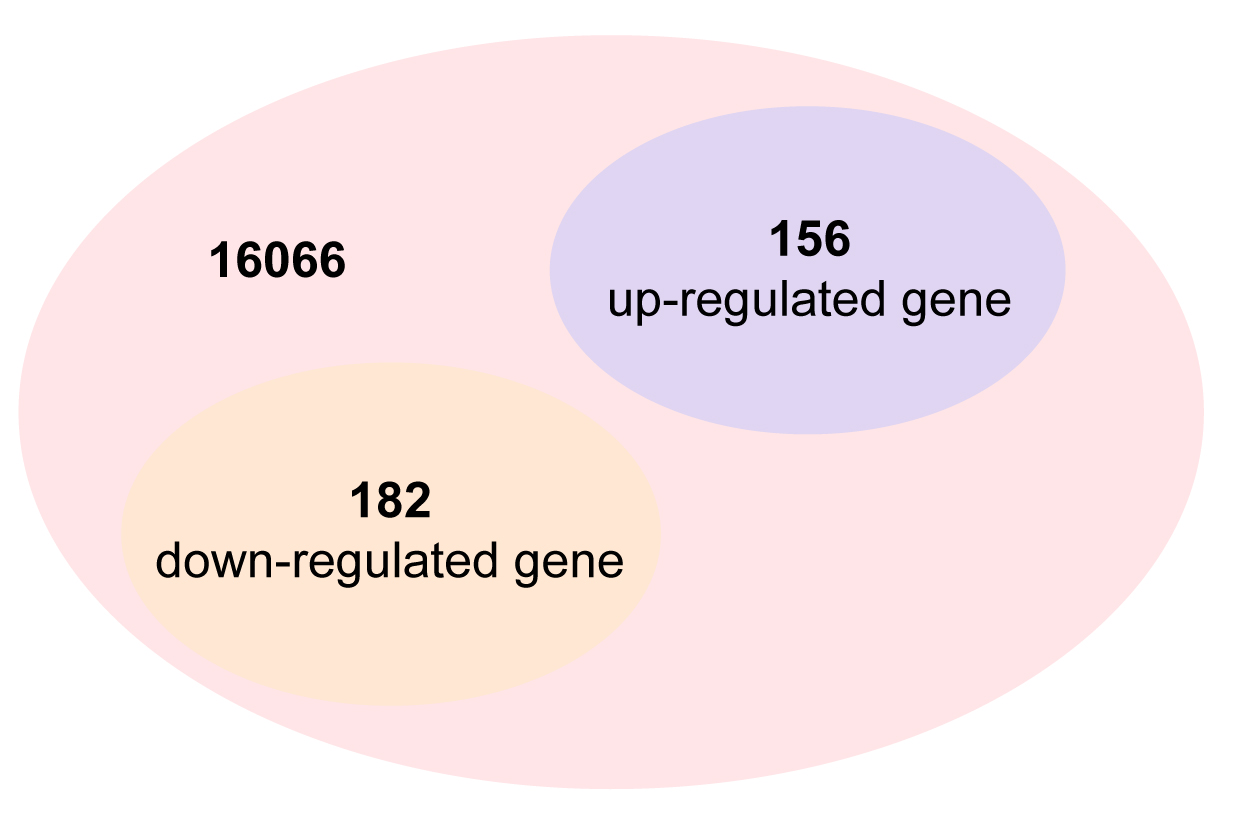


**Fig. S16.** Venn diagram of DEGs.


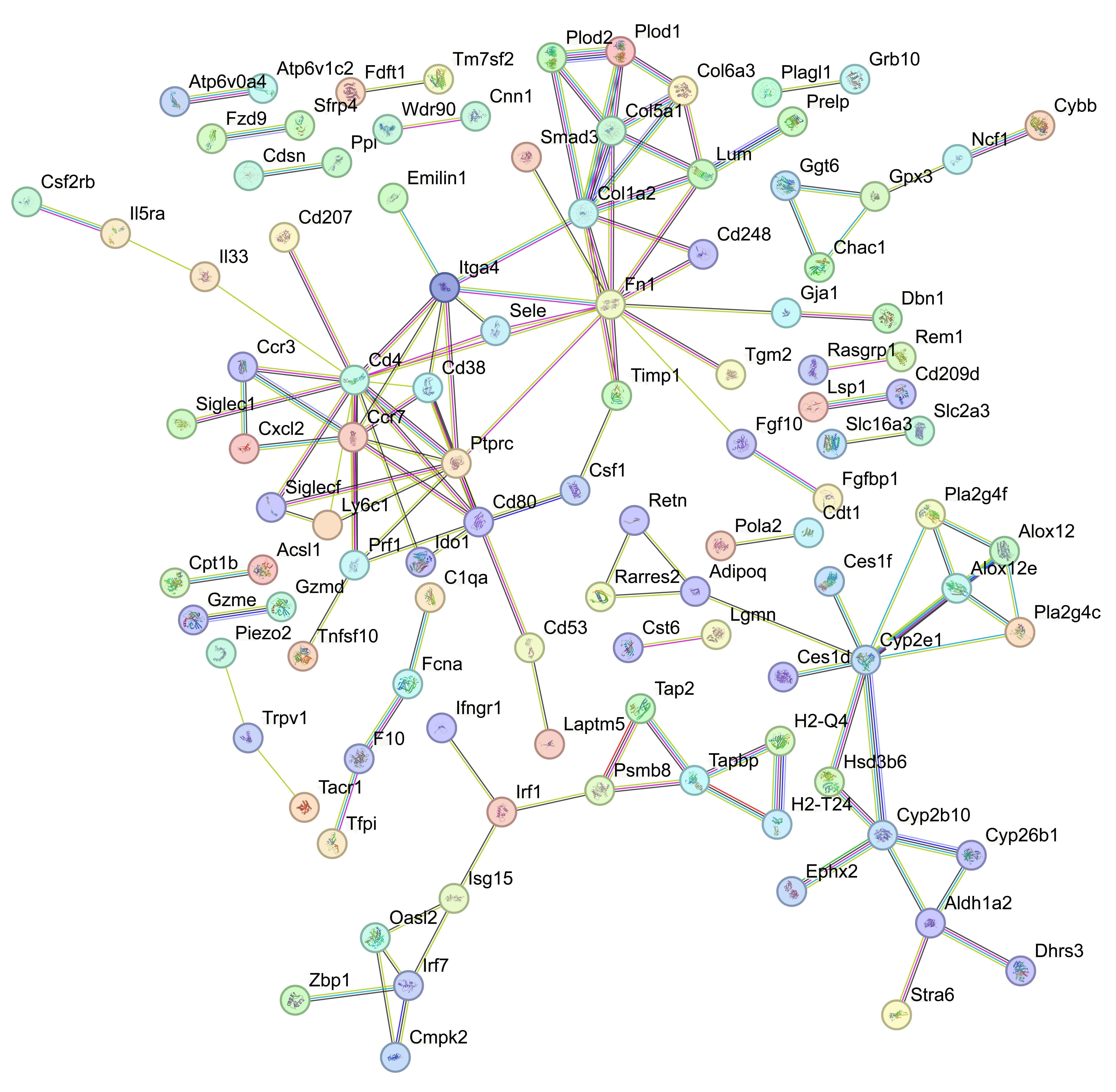


**Fig. S17.** PPI network analysis of differentially expressed genes.
